# Cellular Upcycling of Polyethylene Terephthalate (PET) With an Engineered Human Saliva Metagenomic PET Hydrolase

**DOI:** 10.1002/cssc.202502560

**Published:** 2025-12-08

**Authors:** Rawiporn Amornloetwattana, Bhumrapee Eiamthong, Piyachat Meesawat, Piyakamon Bunkum, Benjamin Royer, Nicoll Zeballos, Marcos Valenzuela‐Ortega, Robert C. Robinson, Stephen Wallace, Chayasith Uttamapinant

**Affiliations:** ^1^ School of Biomolecular Science and Engineering Vidyasirimedhi Institute of Science and Technology (VISTEC) Rayong Thailand; ^2^ Institute of Quantitative Biology Biochemistry and Biotechnology School of Biological Sciences University of Edinburgh Edinburgh UK

**Keywords:** biodegradation, enzyme engineering, metabolic engineering, polyethylene terephthalate, upcycling

## Abstract

Recent advances in biocatalytic recycling of polyethylene terephthalate (PET) using PET hydrolase enzymes have sparked interest in integrating PET degradation capabilities into living systems. Although cell‐based strategies are limited by the mesophilic temperature constraints of microbial hosts, they offer a unique opportunity to couple PET depolymerization with biological upcycling into value‐added chemicals. Here, a comprehensive approach for the cellular degradation and valorization of PET is reported. The crystal structure of MG8, a PET hydrolase identified from the human saliva metagenome is solved, and molecular dynamics simulations are used to pinpoint loop regions for targeted mutagenesis aimed at enhancing activity under moderate temperatures. Over 1000 MG8 loop variants are evaluated with a high‐throughput mass spectrometric screening platform. Two catalytically improved mutants—MG8^G127Y/F250A^ and MG8^N125S/G127Y/F250A^—exhibit significantly enhanced PET hydrolysis at 37°C. To enable whole‐cell PET valorization, a two‐strain *Escherichia coli* system called PETCAT is constructed: one strain is engineered to secrete MG8^G127Y/F250A^ for PET degradation, and the other harbors a synthetic pathway comprising seven heterologous genes for the conversion of terephthalic acid (TPA) into catechol, a versatile intermediate used in pharmaceuticals and fragrances. This study establishes a modular, one‐pot microbial platform for PET recycling and upcycling under physiologically relevant conditions.

## Graphical abstract

To enable whole‐cell PET valorization, PETCAT is constructed as a two‐strain *Escherichia coli* system: one engineered to secrete the human saliva metagenomic PET hydrolase optimized for PET degradation at moderate temperatures; and another harboring a synthetic pathway for the conversion of terephthalic acid, a major PET monomer, into catechol—a versatile intermediate for pharmaceuticals and fragrances.

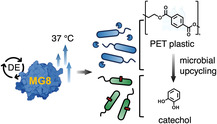

## 1 Introduction

Significant progress toward enzymatic recycling of polyethylene terephthalate (PET) has been made in recent years [1], driven by the approach's distinct advantages that complement mechanical [2] and chemical recycling strategies [3]. Enzymatic PET recycling yields value‐retained monomers comparable to chemical recycling, while offering the lower energy demands and reduced greenhouse gas emissions [4] characteristic of mechanical recycling. Owing to the intrinsic selectivity of PET hydrolases for polyesters, these biocatalytic approaches can also act on mixed plastic feedstocks, potentially reducing the need for costly waste‐sorting operations [5]. Rapid progress in the discovery and engineering of PET‐degrading enzymes [6, 7] has, in turn, enabled advances in feedstock pretreatment, process design for enzymatic plastic depolymerization [8], and recovery of monomers and valuable by‐products, culminating in the first industrial‐scale demonstrations of enzyme‐based PET recycling.

Current enzymatic PET recycling approaches predominantly employ highly active and thermostable PET hydrolases such as engineered variants of leaf‐and‐branch compost cutinase (LCC) [9], Mipa‐P and Kubu‐P [10], PHL7 [11, 12], and *Ideonella sakaiensis* PETase (*Is*PETase) [13, 14]. These enzymes operate near the glass transition temperature of PET (*Tg*, ≈70°C), where enhanced chain mobility in amorphous regions facilitates depolymerization. Recent studies, however, have shown that water acts as a plasticizer for ultrathin PET films, lowering the *Tg* of surface layers in aqueous environments to mesophilic temperatures (40–44°C) [15, 16]. This suggests that efficient PET degradation by mesophilic hydrolases could be feasible when targeting amorphous surface regions under physiological conditions.

In addition to *Is*PETase, various mesophilic and psychrophilic PET hydrolases have been characterized. Several of these hydrolases—including Mors1 from the Antarctic bacteria *Moraxella* sp. Strain TA144 [17, 18], *Ca*PETase from *Cryptosporangium aurantiacum* [19], and a human saliva metagenomic PET hydrolase MG8 discovered by our group [20]—exhibit greater PET hydrolytic activities than *Is*PETase at low to moderate temperatures. Critically, both Mors1 and MG8 likely have greater active‐site flexibility compared to thermophilic hydrolases, a characteristic that enables enzyme adaptation to lower temperatures [21].

Highly active mesophilic PET hydrolases offer unique opportunities for PET recycling and upcycling directly within living cells. Such whole‐cell biocatalysis is inherently cost‐effective, as it eliminates the need for enzyme purification and allows cofactor and energy regeneration to be maintained by cellular metabolism. Integrating PET degradation with microbial metabolism further permits in situ bioconversion of PET‐derived monomers into higher‐value chemicals through native or engineered pathways. The PET monomer terephthalic acid (TPA) can be metabolized via the protocatechuic acid (PCA) pathway in natural or engineered microbes to produce diverse compounds such as gallic acid [22], pyrogallol [22], vanillin [23], muconic acid [22], β‐ketoadipic acid [24], adipic acid [25], and paracetamol [26]. The other monomer, ethylene glycol (EG), can enter central metabolism through the glyoxylate shunt of the Embden‐Meyerhof‐Parnas (EMP) pathway [22]. Most reported PET upcycling examples, however, rely on purified TPA and EG, or on monomers obtained by harsh chemical depolymerization. In one of our previous investigations, exogenous LCC was supplied to degrade PET, and the resulting TPA was converted into vanillin in an engineered *E. coli* strain [23].

Herein, we achieve cellular degradation and upcycling of PET through a sequence of complementary advances. First, we determined the crystal structure of the human saliva metagenomic PET hydrolase MG8 and, guided by molecular dynamics simulations, identified flexible loop region for targeted mutagenesis to enhance activity at moderate temperatures. Second, we developed a high‐throughput mass spectrometric platform to screen over 1000 MG8 loop variants and identified two mutants, MG8^G127Y/F250A^ and MG8^N125S/G127Y/F250A^, with markedly improved PET hydrolysis at 37°C. To enable whole‐cell PET degradation, we engineered an *E. coli* secretion system for MG8^G127Y/F250A^, allowing efficient depolymerization of low‐crystallinity PET. Finally, we coupled these “PET cells” with metabolically engineered “CAT cells” harboring a seven‐gene heterologous pathway that converts TPA to catechol, establishing PETCAT, an integrated cell‐based, two‐strain platform for the one‐pot biological degradation and valorization of PET waste into a versatile chemical feedstock used in pharmaceuticals and fragrances.

## 2 Results

### 2.1 Structural Characterizations Reveal Key Flexible Loops of MG8

**FIGURE 1:**
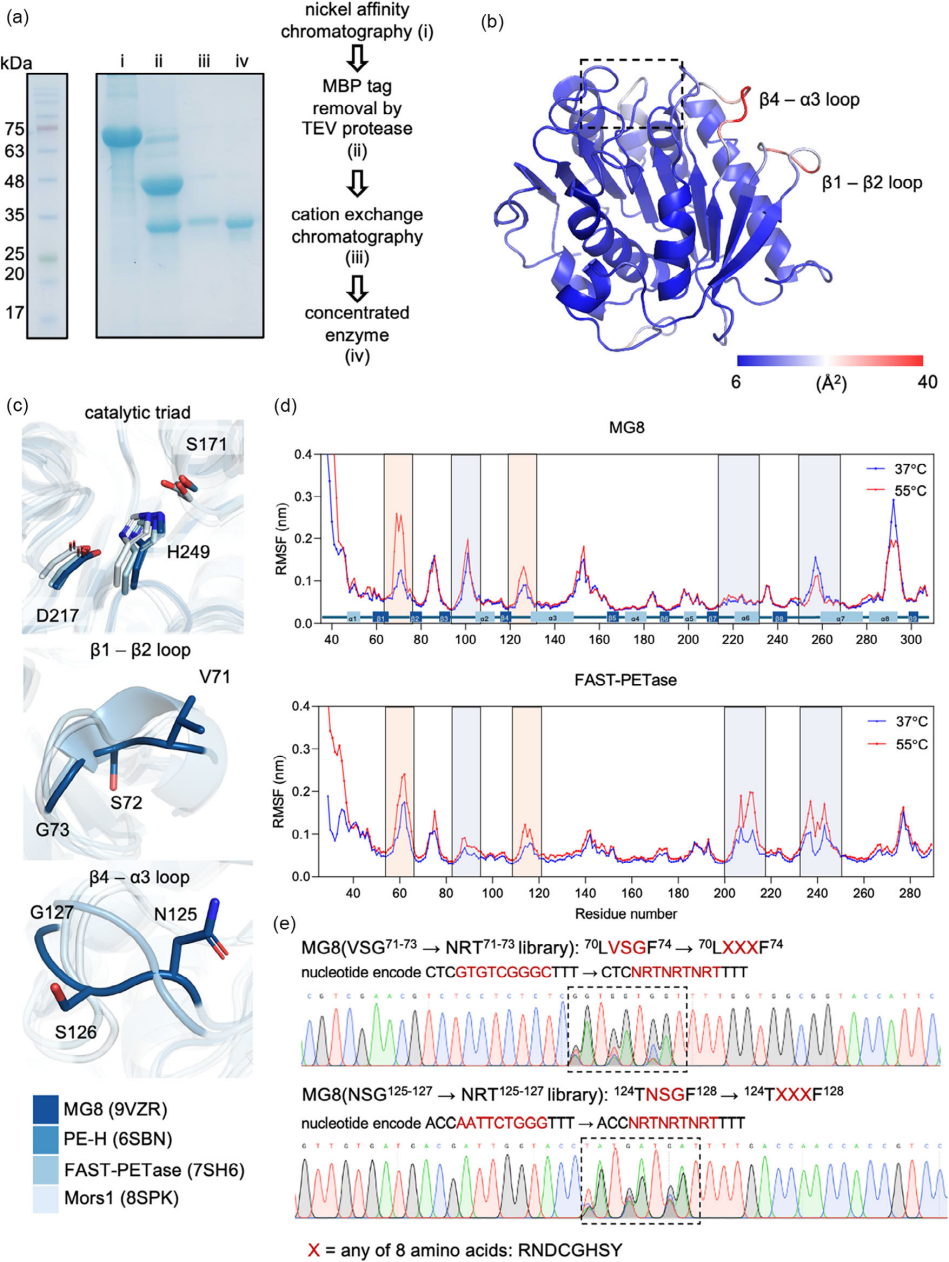
Structural characterization and molecular dynamics simulations of MG8. (a) The protein purification procedure to obtain soluble MG8 for crystallization, and the resulting protein purity. SDS‐PAGE of proteins at each purification step is shown. (b) Crystal structure of MG8. A cartoon representation of MG8 structure at 1.2 Å resolution is shown (Protein Data Bank (PDB) ID code 9VZR). MG8 active site is oriented at the top (dashed rectangle). The cartoon is pseudocolored in blue–white–red according to B‐factors extracted from crystallographic refinement, from minimum (6 Å^2^; blue) to maximum (40 Å^2^; red). β1–β2 and β4–α3 loops showed the highest B‐factors at 35–40 Å. (c) Superimposition of the MG8 structure with those of mesophilic class II PET hydrolases. The catalytic triads (top), β1–β2 loop (middle; secondary structure numbering according to MG8) and β4–α3 loop (bottom) are highlighted. (d) Backbone root mean square fluctuations (RMSF) of MG8 and FAST‐PETase from the last 20 ns of 100 ns simulations under two temperatures. Simulations for both enzymes were performed at 150 mM NaCl. The PET‐capturing loop, the catalytic aspartate loop and the catalytic histidine loop, all of which participate in reaction catalysis, are highlighted in purple. β1–β2 and β4–α3 loops are in orange. (e) Libraries of combinatorial mutations in which nucleotides encoding the three central residues of β1−β2 and β4−α3 loops of MG8 were replaced by the NRT codon. Sequence chromatograms of each library are shown.

We reasoned that achieving a balance between enzyme activity and stability at mesophilic temperatures, particularly between prepaying entropic penalties through active site preorganization [27] and maintaining sufficient structural flexibility for function at low to moderate temperatures [28], could be key to create optimal mesophilic PET hydrolases. We first set out to obtain the crystal structure of MG8 and flexibility information encoded within. We previously found that MG8 majorly partitioned into inclusion bodies upon expression in conventional *E. coli* hosts [2]. To increase yields of soluble proteins, we genetically fused MG8 to the C‐terminus of highly soluble maltose‐binding protein (MBP), with a TEV cleavage site between MBP and MG8. The fusion enzyme could be expressed in soluble forms in *E. coli* BL21(DE3) and purified in three steps using Ni‐NTA affinity chromatography, TEV protease‐mediated cleavage, and cation exchange chromatography to afford soluble MG8 at a yield of ≈5 mg/L culture, and a stock concentration of ≈16 mg/mL (Figure 1a and S1).

Crystallization trials of MG8 were performed using the sitting drop vapor diffusion technique and the PACT premier screen kit; rectangular prism crystals were obtained under 100 mM NaOAc pH 5.0 containing 200 mM NaCl and 20% v/v PEG6000. The crystal structure of MG8 (P2_1_2_1_2_1_ space group) was solved and refined to the resolution of 1.2 Å (Figure 1b and S2; refinement statistics in Table S1). The structure showed a typical α/β‐hydrolase fold, a canonical catalytic triad (S171, D217, H249), two stabilizing disulfide bonds (C214‐C251 and C288‐C305) typical of class II PET hydrolases, and high structural homology to other known mesophilic class II enzymes such as FAST‐PETase [29], Mors1 [11], and PE‐H [30] (Figure 1c and S2b). MG8's unique extended catalytic histidine loop between β‐strand 8 and α‐helix 7 shown to be critical for its mesophilic activity [20] was well‐resolved in the structure. Of all regions of MG8 in the structure, the β1–β2 and β4–α3 loops exhibited the highest flexibility, with atomic B factors up to 40 Å^2^ (Figure 1b). Neither loop is expected to contribute via direct contact toward PET binding nor catalytic steps. Comparison of the three central residues of β1–β2 and β4–α3 loops (VSG^71−73^ and NSG^125−127^, respectively) in analogous loops from other mesophilic class II PET hydrolases showed distinct conformations these loops can adopt in different crystal structures (Figure 1c, middle and bottom), pointing to the overall high local flexibility of the two loops across mesophilic PET hydrolases. The loop flexibility is in contrast with more rigidly positioned catalytic triad residues across PET hydrolases (Figure 1c, top).

### 2.2 Molecular Dynamics (MD) Simulations of MG8 Further Confirm Potential Engineering Sites

We further performed MD simulations to assess structural dynamics of MG8 at short timescales (100 ns), the information of which could complement experimentally observed structural dynamics in the crystal structure. The simulations for MG8 were conducted at 150 mM NaCl and two temperatures (37 and 55°C); 55°C was previously shown to be the optimal operating temperature of MG8 [20] (Figure 1d). We also performed simulations for the state‐of‐the‐art engineered mesophilic PET hydrolase FAST‐PETase [29], whose enhanced activity was previously shown via simulations to be linked to increased flexibility of its Asp catalytic loop [31].

As demonstrated by the root mean square fluctuations (RMSF, Figure 1d), the catalytic histidine (β8–α7) loop for both MG8 and FAST‐PETase across both NaCl and temperature conditions exhibited high flexibility. In contrast, there seemed to be differences in flexibilities of the catalytic aspartate (β7–α6) loops of the two enzymes: the flexibility of this catalytic loop is high for FAST‐PETase but low for MG8. The loop flexibility trend is interestingly switched for the β3–α2 loop proposed as key to PET substrate capture [32]: the flexibility of the β3–α2 loop is high for MG8 but low for FAST‐PETase. Such a trade‐off in the flexibility of the catalytic aspartate loop versus the β3–α2 PET‐capturing loop, particularly the pairing of the low‐flexibility catalytic aspartate loop with the high‐flexibility β3–α2 loop of MG8, has been proposed as critical for PET hydrolase activity enhancement observed in HOT‐PETase [14]. Despite the difference in flexibility trends predicted by MD in the catalytic (β7–α6) and substrate binding (β3–α2) loops between MG8 and FAST‐PETase, we reasoned that these loop regions of MG8 are sufficiently optimal as the preorganization of the enzyme active site is compensated by the increased flexibility of the PET‐capturing loop.

The β1–β2 and β4–α3 loops with high atomic flexibility in the MG8 crystal structure (Figure 1b) showed ΔRMSF of 1.5 and 0.5 Å, respectively, when the simulation temperature was shifted from 37 to 55°C (Figure 1d). The increase in loop flexibility at higher temperatures may cause the enzyme to become unstable and lose activity. However, at low or moderate temperatures, it could reduce the enthalpic penalty associated with conformational changes needed to reach the transition state. Previous studies have established links between catalytic rates and the flexibility of loops outside of the active site [33]. In particular, a comparison study between cold‐ and warm‐adapted trypsins shed light to the roles of point mutations in loop regions distal from the active site in influencing the activation enthalpic‐entropic balance through changes in the softness of protein surface [34]. We thus thought that tuning conformational dynamics of the β1–β2 and β4–α3 loops could be an effective approach in creating more active MG8 variants at mesophilic temperatures.

### 2.3 High‐Throughput Mass Spectrometric Screening of Loop Mutants of MG8 for Better Mesophilic Activity

We created two libraries of combinatorial mutations at the three central residues of β1–β2 and β4–α3 loops (VSG^71−73^ → NRT^71−73^ library; and NSG^125−127^ → NRT^125−127^ library respectively; cloning primers in Table S2) of MG8. As site‐saturation mutagenesis with NNK/NNS codons would generate too large libraries for our mass spectrometry‐based screening platform, we used a more focused NRT degenerate codon, which encodes a set of eight amino acids representing the four core biophysical properties (nonpolar Cys and Gly; polar Ser, Tyr, and Asn; basic Arg and His; and acidic Asp) and no stop codon. Combinatorial mutations with the NRT codon at the three residues of each loop would theoretically generate a library of 512 variants. In addition, we previously reported the F250A mutant of MG8 with enhanced hydrolytic activity on small PET oligomers while preserving good hydrolytic activity on solid PET [20], and decided to use MG8^F250A^ as the template to generate the two loop libraries. Sequencing showed appropriate sequence diversity at mutagenized positions of the two libraries (Figure 1e and S3).

**FIGURE 2:**
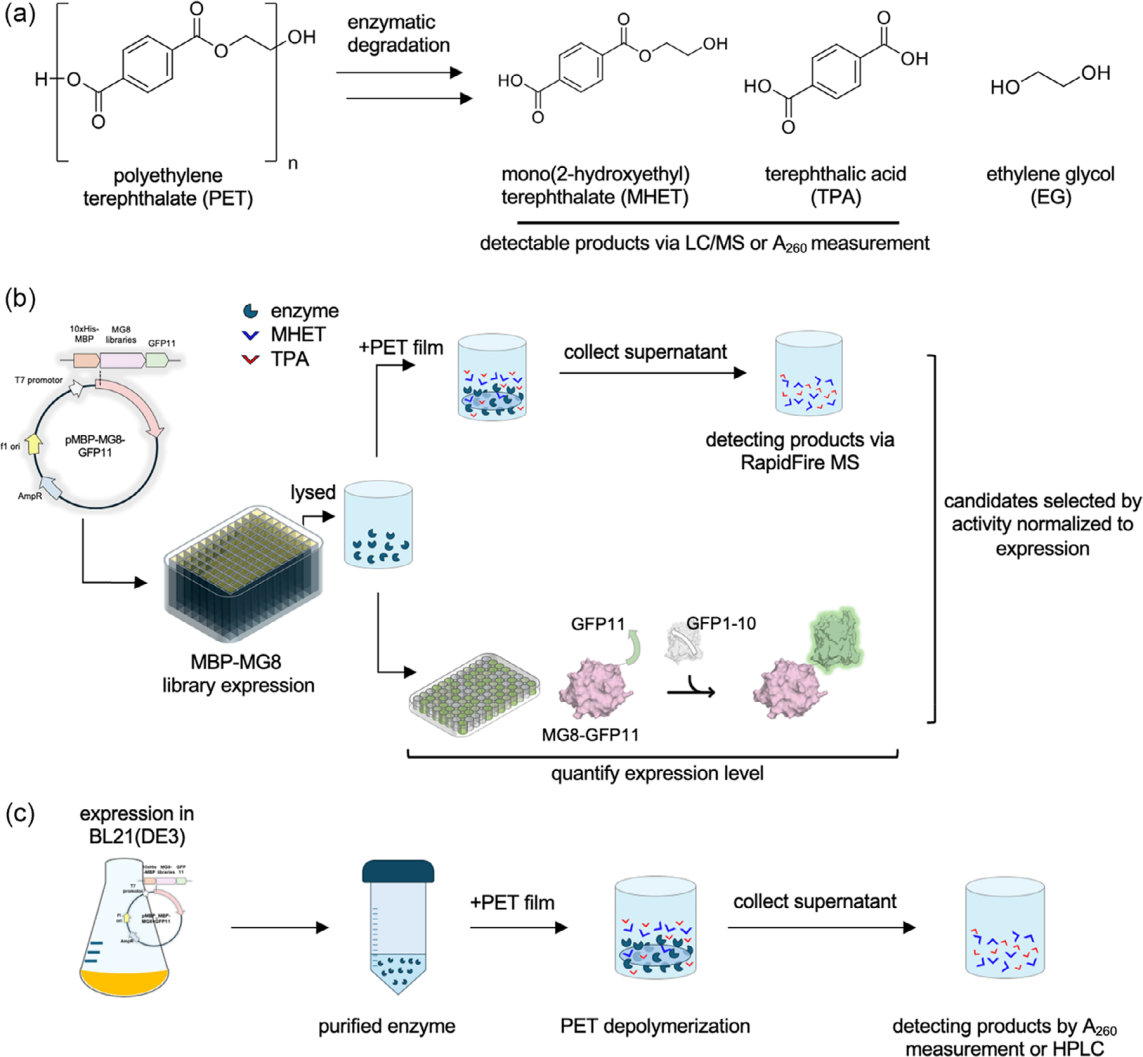
Workflow for the mass spectrometry‐based screening of enzymatic PET hydrolysis activity. (a) Enzymatic PET degradation reaction. Mono(2‐hydroxyethyl) terephthalate (MHET) and terephthalic acid (TPA) generated from the degradation of PET can be monitored in a high‐throughput manner by a RapidFire mass spectrometer (MS), or by HPLC. Release of soluble aromatic content (MHET + TPA) can also be measured by changes in absorbance at 260 nm. (b) Workflow for normalized activity measurements in 96‐well plates. BL21(DE3) expressing MBP‐ and GFP11‐tagged MG8 libraries were lysed, and the cell lysates used in parallel for MG8‐mediated PET degradation reactions (top) and MG8 expression level measurements (bottom). Top, lysates were incubated with PET film for 2 days at 37°C or 55°C. Degradation products (MHET and TPA) were then quantified with the RapidFire MS. Bottom, lysates were incubated with GFP1−10 to reconstitute split GFP. GFP fluorescence intensities were then measured on a microplate reader. Enzyme candidates for superior PET degradation were selected based on PET degradation activity normalized with their expression levels. (c) Selected candidates were expressed and purified from BL21(DE3). Relative activity of purified enzymes in hydrolyzing PET film was characterized by A_260_ measurements or by HPLC.

We developed a screening workflow to assess enzymatic PET hydrolysis activity (Figure 2a) of MG8(NRT^71−73^/F250A) and MG8(NRT^125−127^/F250A) libraries based on the high‐throughput RapidFire 365 mass spectrometry system (Figure 2b). Each library was transformed into *E. coli* BL21(DE3) and ≈1250 individual colonies per library, representing >90% experimental coverage of the theoretical library size, were used to inoculate 400 μL of LB media, after which MG8 variant overexpression was induced. Enzyme variants were evaluated for PET hydrolysis activity in crude cell lysates prepared with BugBuster in 96‐well plates, using low‐crystallinity (9%) PET film as the substrate (Figure S4; PET crystallinity characterizations in Table S3 and Figure S5). As we included the GFP11 peptide tag at the C‐terminus of MG8 variants, their relative expression levels in crude lysates could be assessed with split GFP reconstitution [35] and performed in parallel with activity measurements.

We assessed enzymatic PET hydrolysis activity through quantification of PET degradation products mono(2‐hydroxyethyl)terephthalate (MHET) and terephthalic acid (TPA) on RapidFire mass spectrometry (MS). Commercial TPA and enzymatically generated MHET [20] spiked in cell lysates were first used as standards for MS method optimizations, where optimal dilutions of cell lysates to minimize ion suppression were determined (Figure S6). We used a single retention time to extract total negative ions, from which the mass‐to‐charge ratios (m/z) of interest corresponding to the parent ions of MHET (m/z 209.0) and TPA (m/z 165.0) and two MRM transitions of MHET and TPA (MHET: m/z 165.0 and 121.0; TPA: m/z 121.0 and 77.0) could be observed (Figure S7). First‐order daughter ion counts could then be correlated to PET degradation product amount in the samples. Measurements of all products at the same retention time greatly sped up screening, enabling us to characterize a 96‐well plate within ≈2 h, and a given loop library with replicates in ≈1 week. Subsequently, MHET and TPA daughter ion peak areas were normalized with reconstituted GFP fluorescence intensities to yield normalized PET hydrolysis activity levels for each MG8 loop variant.

We performed activity screening of MG8(NRT^71−73^/F250A) and MG8(NRT^125−127^/F250A) libraries at two temperatures: 37°C and 55°C. In general, we expectedly observed more MHET produced at 37°C, while MHET‐mediated product inhibition [36] was largely relieved at 55°C, resulting in more TPA produced. We selected the top 30 variants with consistently high activity levels at 37°C for rephenotyping in the same lysate‐based setup at both temperatures and sequencing (Table S4‐5). Among the mutated positions, we found G73 of the β1β2 loop and S126 of the β4–α3 loop remained largely conserved.

### 2.4 Efficient PET Plastic Degradation by MG8^G127Y/F250A^ and MG8^N125S/G127Y/F250A^ at 37°C

To ensure that our comparison was not biased by the activity assay context within lysates, we proceeded with the expression and purification of the top 15 variants for further characterization in a PET degradation assay with purified enzymes (Figure 2b). We constructed expression plasmids for the top 15 MG8 variants with an N‐terminal MBP tag for increased protein solubility (Figure S8). PET degradation activity was tested on PET film (6 mm diameter; 9% crystallinity) in 25 mM glycine pH 9 buffer and a range of NaCl concentrations (0.01–4 M), then produced MHET and TPA assessed under HPLC.

**FIGURE 3:**
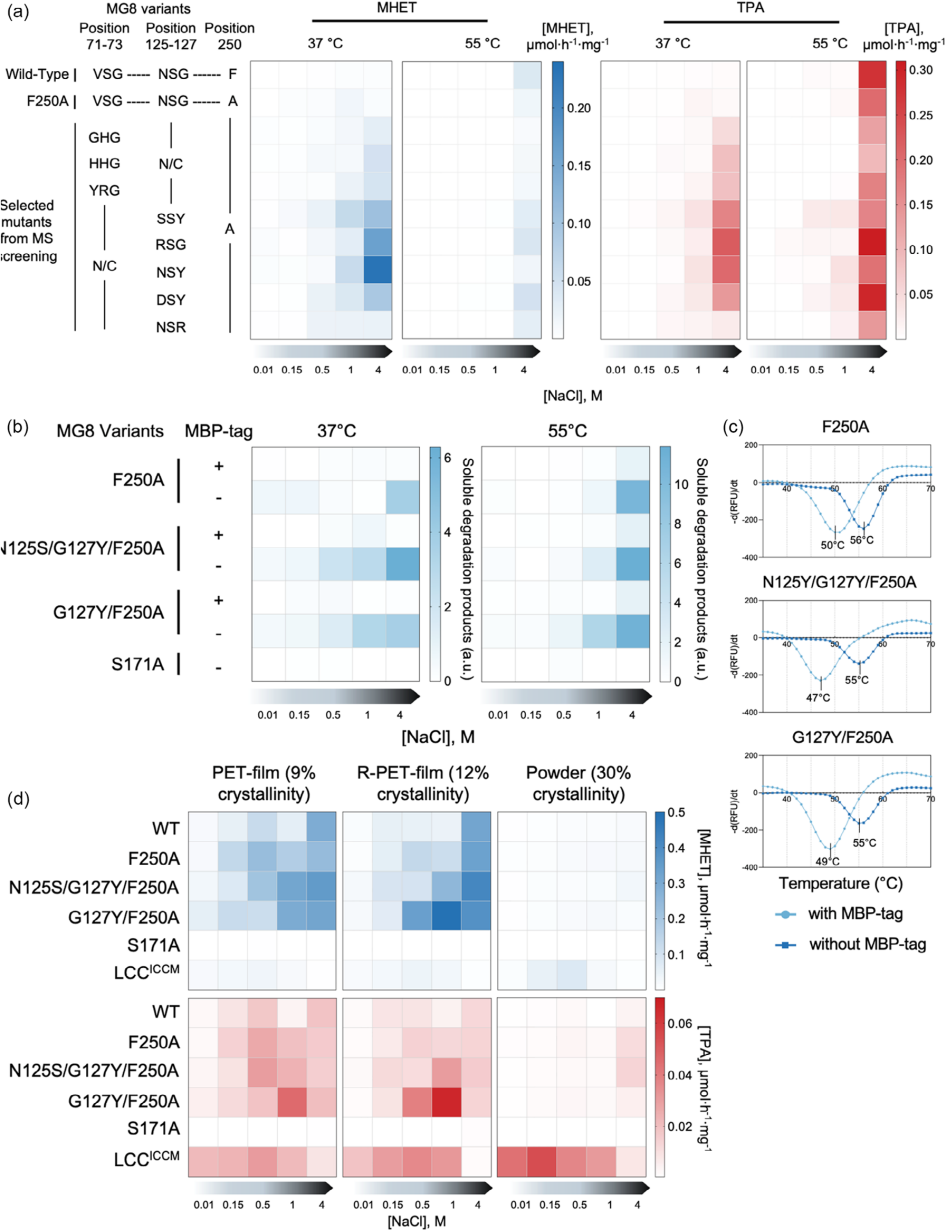
PET degradation activity by selected MG8 variants. (a) Relative activity of eight purified MBP‐tagged MG8 variants from the β1–β2 and β4–α3 loop libraries in hydrolyzing PET film to generate MHET (left) and TPA (right). Hydrolysis was performed for 2 days under different NaCl concentrations (0.01–4 M) and temperatures (37°C and 55°C) before quenching with formic acid and assessed by HPLC. MBP‐tagged wild‐type MG8 and MG8^F250A^ act as reference points. Each entry in the heat map represents mean values from triplicate experiments. (b) Superior activities of MG8^G127Y/F250A^ and MG8^N125S/G127Y/F250A^ are maintained after MBP tag removal. PET degradation reactions were set up as in **a**, then soluble PET degradation products assessed by absorbance measurements at 260 nm. MG8^S171A^ has its catalytic serine mutated and is mechanistically inactive. Heat maps show mean amount of products generated in triplicate experiments. (c) Thermal stabilities of MG8^G127Y/F250A^ and MG8^N125S/G127Y/F250A^ with MG8^F250A^ as a reference point. (d) Relative activity of MG8 variants in degrading different PET substrates at 37°C. Assays were set up as in **a** with 7–10 mg of different PET substrates. The ICCM quadruple mutant of the leaf‐and‐branch compost cutinase (LCC) was used as an activity reference point. Heat maps show mean amount of MHET and TPA generated in triplicate experiments. For bar graphs of a, b, and d with error bars, see Figures S9, S10, and S13.

We obtained eight promising MG8 variants (Figure 3a and S9). While more active mutants compared to wild‐type MG8 and MG8^F250A^ emerged from both NRT^71−73^ and NRT^125−127^ libraries, the best mutants from the NRT^125−127^ library were more active than those from the NRT^71−73^ library (≈2–5‐fold more active at 37°C; ≈2–10‐fold more active at 55°C; assessed from combined MHET and TPA produced). These active variants contained 1–2‐residue changes at the loops, with positions 71–72 of the β1–β2 loop and 125 and 127 of the β4–α3 loop being hotspots for activity‐increasing mutations. Engineered MG8 variants remained highly active at high (1–4 M) NaCl concentrations likely due to their hypersaline environment origins [20].

Among MG8(NRT^125−127^/F250A) highly active variants, MG8^G127Y/F250A^ and MG8^N125S/G127Y/F250A^ emerged as the most active variants at 37°C, with 20–30‐fold higher PET degradation activities than MG8^F250A^ at 4 M NaCl (0.43 µmol_MHET + TPA_.h^−1^.mg_enzyme_^−1^ and 0.27 µmol_MHET + TPA_.h^−1^.mg_enzyme_^−1^, and 0.015 µmol_MHET + TPA_.h^−1^.mg_enzyme_^−1^, respectively, Figure 3a). They also maintained decent activity at lower salt concentrations and at 55°C. We proceeded to remove the MBP tag (which could suppress PET hydrolytic activity of these enzymes) and reassessed activity of MG8^G127Y/F250A^ and MG8^N125S/G127Y/F250A^ in comparison to MG8^F250A^ and inactive MG8^S171A^ (Figure 3b and S10). Here, we used absorbance measurements at 260 nm to determine the total amount of soluble PET degradation products [37]. We found that MBP‐less MG8^G127Y/F250A^ and MG8^N125S/G127Y/F250A^ still maintained excellent PET hydrolysis activity in comparison to MG8^F250A^ (≈70‐fold more active than MG8^F250A^ at 37°C and 1 M NaCl). Their improvements at lower salt concentrations and at 55°C were also preserved. We characterized the thermal stability of MG8^G127Y/F250A^ and MG8^N125S/G127Y/F250A^ in both MBP‐containing and MBP‐less forms and found the *T*_*m*_ of these newly engineered MG8 variants to slightly decrease compared to MG8^F250A^ (Figure 3c and S11).

We suspected the stability‐activity trade‐off, in which a minor increase in instability within the engineered loops helps promote enzymatic activity at mesophilic temperatures, plays a role here. We performed MD simulations to assess dynamics of AlphaFold [38]‐predicted structures of MG8 bearing the G127Y single mutation, and the N125S/G127Y double mutation (Figure S12). Interestingly, these point mutations to the β4–α3 loop seemed to increase dynamics at the catalytic aspartate and histidine loops instead of locally at the mutated loop. The increase in active site flexibility was previously observed in the mesophilic Mors1 PET hydrolase [17] and may also explain the MG8 mutants’ higher activities at moderate temperatures, albeit at compromised thermal stabilities. Further protein engineering can likely help recover or improve the stability of these MG8 variants while preserving current increased activity levels.

### 2.5 Challenges of Mesophilic MG8 Variants in Dealing with Highly Crystalline PET

We tested the ability of our engineered mesophilic MG8 variants in degrading different PET substrates and postconsumer products. We selected three PET substrates to test for enzymatic degradation at 37°C followed by HPLC assessment of degradation products: PET from coffee cup (6 mm diameter; 9% crystallinity), recycled PET food packaging (6 mm diameter; 12% crystallinity); and PET powder (2 mm diameter, 30% crystallinity). We found that while MG8^G127Y/F250A^ and MG8^N125S/G127Y/F250A^ can handle low‐crystalline PET well at 37°C, their efficiency became much reduced when high‐crystalline PET was used (Figure 3d and S13). This is in contrast with the state‐of‐the‐art PET hydrolase LCC^ICCM^ which can degrade PET even at high crystallinity and a moderate temperature to a considerable extent. Nevertheless, under specific reaction conditions, particularly at high ionic strength (4 M NaCl), our engineered enzymes outperform FAST‐PETase and LCC^ICCM^ in degrading low‐crystalline PET at 37°C (Figure S14). At pH 9.0 and 4 M NaCl, MG8^G127Y/F250A^ exhibited an activity of 1.50 µmol_MHET + TPA_.h^−1^.mg_enzyme_^−1^, corresponding to ≈25‐fold and ≈threefold higher rates than FAST‐PETase (0.06 µmol_MHET + TPA_.h^−1^.mg_enzyme_^−1^) and LCC^ICCM^ (0.41 µmol_MHET + TPA_.h^−1^.mg_enzyme_^−1^), respectively.

Under high salt conditions, we further found that addition of 10% v/v DMSO could enhance enzymatic PET degradation of low‐crystallinity PET (in both powder and film forms) by MG8^G127Y/F250A^ by ≈twofold (Figure S15a). Decreasing PET particle size from 6 mm to 0.25 mm diameter through ultracentrifugal milling also helped accelerate product release (reaching 5 AU of A260 measurements of combined product release in 24 h with 0.25 mm particle, compared to 48 h with 6 mm particle, Figure S15b).

### 2.6 Engineered MG8 can be Efficiently Secreted From *E. coli*

Although MG8^G127Y/F250A^ and MG8^N125S/G127Y/F250A^ exhibit improved activity over the parent MG8 in degrading PET at mesophilic temperatures, their moderate thermal stability may limit their applicability in purified enzyme‐based PET recycling processes, which typically require robust performance at elevated temperatures. Moreover, the requirement of high salt (0.5–4 M) concentrations for activity of MG8 variants can complicate process development. On the other hand, cells are known to thrive in conditions with varying salt concentration preferences. *E. coli*'s natural habitat of the mammalian gut has a NaCl concentration of ≈0.1 M but the organism can be adapted for growth at halophilic conditions even up to 1 M NaCl [39, 40]. Typical bacterial growth media such as LB contain ≈0.2 M NaCl and ≈0.4 M osmolarity when taking into account other nondissociating solutes [41]. We thus wondered if the production, secretion and activity of engineered MG8, which functions well within salinity ranges compatible with *E. coli* growth, could be optimized while keeping the organism viable to create a cell‐based degradation platform for PET.

**FIGURE 4:**
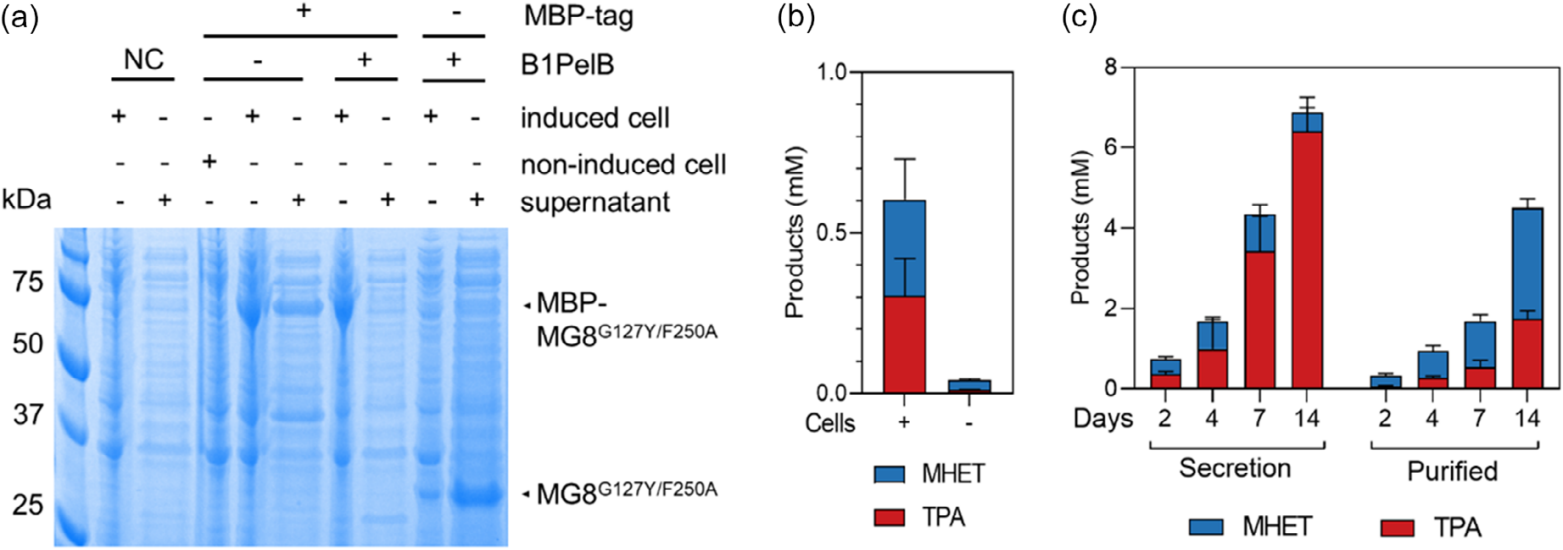
Cell‐based PET degradation with secreted MG8^G127Y/F250A^ **.** (a) The B1PelB signal sequence mediated secretion of MG8 variants into the extracellular media. SDS‐PAGE of total proteins in cell pellets versus in the cell growth media are shown. (b) PET degradation by MG8^G127Y/F250A^ in the extracellular media, in the presence versus absence of the *E. coli* secretor cells. BL21(DE3) was transformed with the expression plasmid for B1PelB‐MG8^G127Y/F250A^ and enzyme expression induced for 66 h at 25°C. 500 µL of the culture versus cell‐free supernatant, each containing ≈3.3 µM secreted MG8^G127Y/F250A^, was then supplemented with 50 mM K_2_HPO_4_/KH_2_PO_4_ pH 8.0, 0.5 M NaCl and 5% v/v DMSO and used to hydrolyze 7 mg PET powder at 37°C for 2 days before quenching with PMSF. Generated MHET and TPA were then analyzed by HPLC. Error bars, ± S.D. from triplicates. (c) Cell‐based PET degradation with secreted MG8^G127Y/F250A^ versus degradation with purified MG8^G127Y/F250A^. For purified enzyme‐based degradation, 3 µM MG8^G127Y/F250A^ was used to hydrolyze 7 mg PET powder in 50 mM K_2_HPO_4_/KH_2_PO_4_ pH 8.0, 0.5 M NaCl and 5% v/v DMSO at 37°C for 2–14 days before quenching with PMSF. For cell‐based degradation, BL21(DE3) cells were transformed and enzyme expression induced for 66 h at 25°C as in **b**. 500 µL of the culture containing ≈3 µM secreted MG8^G127Y/F250A^ was then supplemented with 50 mM K_2_HPO_4_/KH_2_PO_4_ pH 8.0, 0.5 M NaCl and 5% v/v DMSO and used to hydrolyze 7 mg PET powder at 37°C for 2–14 days before quenching. Generated MHET and TPA were then analyzed by HPLC. Error bars, ± S.D. from triplicates. Secreted MG8^G127Y/F250A^ concentrations before supplementation of PET powder for **b** and **c**: 105 and 104 μg/mL, respectively.

We first established a protein secretion system for engineered MG8. We replaced MG8's native signal peptide [20] with the B1PelB signal sequence [42], which combines a signal peptide enhancer B1 with the commonly used Sec‐dependent signal peptide from *Erwinia carotovora* pectate lyase B (PelB). The use of B1 enhancer is previously shown to overcome periplasmic trapping of proteins if PelB alone was used, and helps enhance extracellular secretion [42]. Initial empirical optimizations indicated that B1PelB‐MG8^G127Y/F250A^ could be secreted at higher levels than B1PelB‐MG8^N125S/G127Y/F250A^, presumably due to the former's higher stability (Figure S16); we therefore carried out subsequent tests with secreted MG8^G127Y/F250A^. Efficient secretion of MBP‐MG8^G127Y/F250A^ and MG8^G127Y/F250A^ to the extracellular media was strictly dependent on the B1PelB sequence presence (Figure 4a). We empirically optimized cell growth and secretion conditions, including culture temperature, expression time, IPTG concentration for induction, and media composition (Figure S17). We found optimal conditions to produce secreted MG8^G127Y/F250A^ to be at 25°C for 66 h in which we obtained ≈119 μg of total protein (the majority of which is the desired enzyme) per mL culture (Figure S16). Secreted MG8^G127Y/F250A^ could be easily purified via nickel affinity chromatography to obtain the protein at a reasonably high yield of ≈60 mg/L culture, and >95% purity (Figure S18).

### 2.7 Cell‐Based PET Degradation with Engineered MG8

We optimized cell‐compatible PET degradation conditions with secreted MG8^G127Y/F250A^ and low‐crystallinity (9%) PET powder at 37°C. Taking into account the high degradation efficiency by MG8 previously observed under basic pH (8–9), moderate‐to‐high [NaCl] (0.5–4 M), and the presence of DMSO, we focused our optimizations on these parameters (Figure S19). The optimal condition for PET powder degradation was found to be the modified M9 media (0.5% w/v glucose, 1% v/v glycerol, and 0.5% w/v yeast extracts) supplemented with 50 mM K_2_HPO_4_/KH_2_PO_4_ pH 8.0, 0.5 M NaCl and 5% v/v DMSO; the condition produced fourfold more degradation products compared to when the degradation was performed in the media without the supplement (up to 0.6 mM MHET + TPA released; Figure S19). *E. coli* cell growth was minimally affected under this supplemented media condition up to 28 h (Figure S20).

Cell‐based PET degradation with secreted MG8^G127Y/F250A^ was then benchmarked against two alternative setups. First, we compared the setups where cells were allowed to secrete engineered MG8 over 3 days, after which the resulting extracellular media was used to degrade PET for additional 2 days, in the presence and absence of the *E. coli* secretor cells. We found that despite the relatively small increase in the concentration of MG8^G127Y/F250A^ in the continued presence of cells (22% increase from 105 to 128 μg/mL after 2 days, Figure S21), we observed 17‐fold improved PET degradation in the presence of cells (Figure 4b). The boost in PET degradation likely stemmed from continual regeneration of fresh and active MG8 from cells, compared to unfolded and/or potentially product‐inhibited MG8 in the absence of cells.

Second, we evaluated cell‐based PET degradation against conventional biocatalytic PET degradation with purified PET hydrolases. The comparison was done over different reaction times (2–14 days) under the optimal degradation condition for MG8^G127Y/F250A^ (50 mM K_2_HPO_4_/KH_2_PO_4_ pH 8.0, 0.5 M NaCl and 5% v/v DMSO at 37°C). Cell‐secreted MG8^G127Y/F250A^ consistently outperformed purified MG8^G127Y/F250A^ and produced 2‐3‐fold more degradation products at all time points, reaching 6.4 mM TPA produced after 14 days (Figure 4c; Figure S22 for secreted enzyme concentrations over time), and yielding ≈9% depolymerization of PET (Table S6). Cell‐secreted MG8 also produced more TPA over MHET compared to purified enzymes, again likely due to continuous enzyme production—which peaked after 2 days of expression at 37°C and remained stable for up to 7 days (Figures S23 and S24)—and effective relief of product inhibition. MHET‐mediated inhibition of PET hydrolases is known to be relieved at high temperatures (>50°C) or with the use of lidded carboxyesterases such as *Thermobifida fusca* KW3 TfCa [36] and *I. sakeiensis* MHETase [14]. Here we showed that MHET‐inhibition of a PET hydrolase could be effectively overcome at 37°C simply by enzyme regeneration from cells.

### 2.8 Concomitant Cell‐Based PET Degradation and Upcycling to Catechol

**FIGURE 5:**
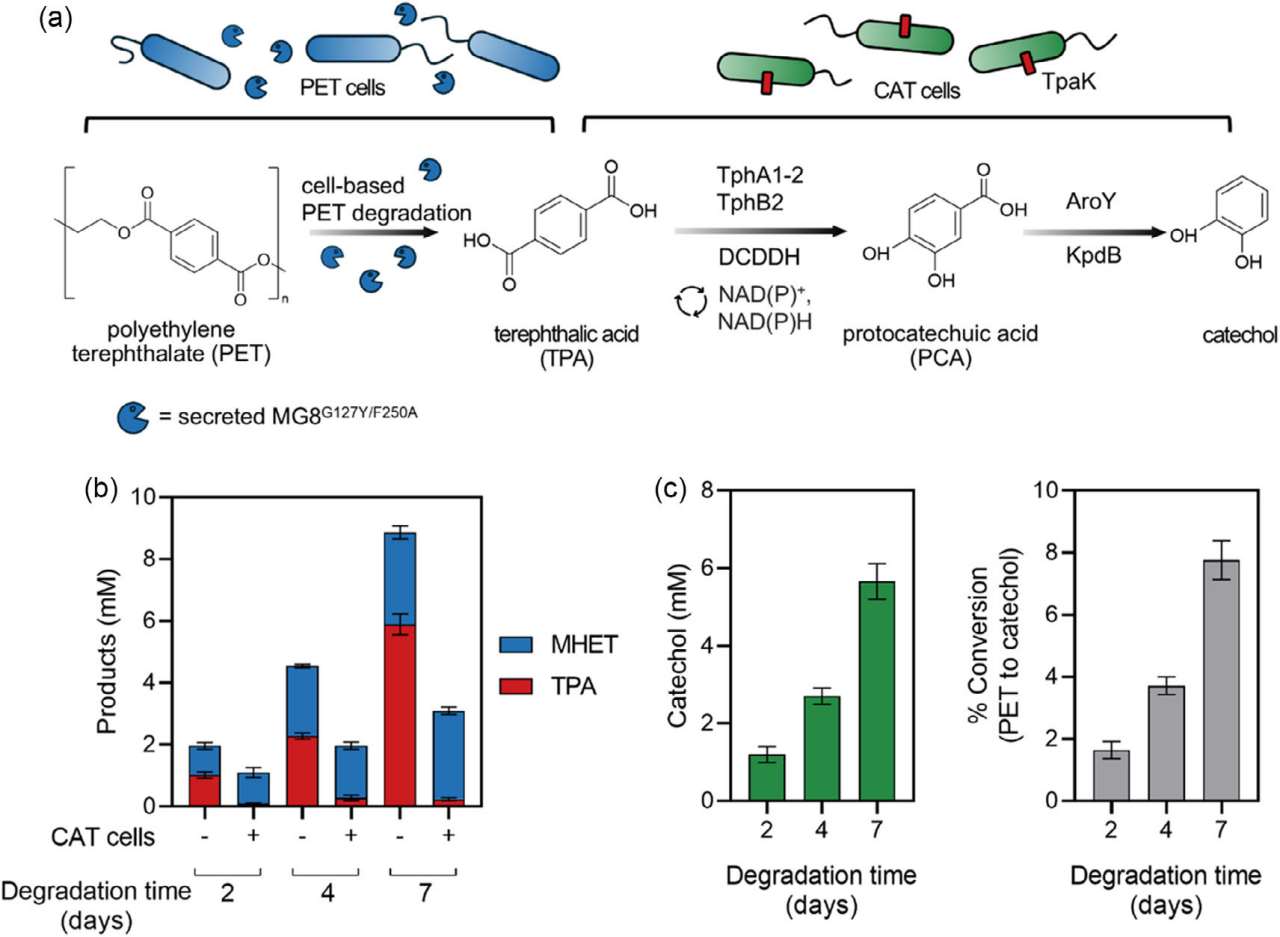
Concomitant cell‐based PET degradation and upcycling to catechol. (a) PET‐degrading *E. coli* secreting MG8^G127Y/F250A^, or PET cells, were first used to degrade PET. Thereafter, TPA‐to‐catechol‐converting *E. coli* or CAT cells were added. The engineered biosynthetic pathway to generate catechol from TPA is shown. (b) Generated MHET and TPA from PET degradation for 2, 4, and 7 days prior to CAT‐cell addition and after 2 days of incubation with CAT cells. (c) Catechol bioconversion from produced TPA after CAT‐cell addition for 2 days. Generated catechol concentrations (mM, left) and percent conversion to catechol relative to PET amount (right) are shown. Error bars, ± S.D. from triplicates. Secreted MG8^G127Y/F250A^ concentrations before supplementation of PET powder: 39 μg/mL.

We previously reported an optimized TPA degradation pathway via heterologous expression of six genes in *E. coli* for the conversion of TPA to catechol [25] (Figure 5a). The TphA1−2 and TphB2 TPADO dioxygenase and DCD dehydrogenase, all from *Comamonas* sp., collectively perform oxidative decarboxylation of TPA to protocatechuate (PCA). PCA is then transformed into catechol via a PCA decarboxylase AroY and its prenylated FMN‐generating activator KpdB, both from *Klebsiella pneumoniae*. Here we added the seventh component, a TPA transporter TpaK from *Rhodococcus jostii* and combined all seven genes into two expression plasmids: pPCA4‐*tpado*‐*dcddh*‐*tpaK* and pQlinkN‐*aroY‐kpdB*. TPADO subunits and DCDDH were expressed under the IPTG‐inducible Ptac promoter; TpaK transporter under the BBa_J23106 constitutive promoter; and AroY and KpdB under the T5 promotor. Both plasmids were transformed into *E. coli* BL21(DE3) to produce TPA‐to‐catechol‐converting or CAT cells (Figure 5a).

Microbial transformation of TPA to catechol with CAT cells was first validated and optimized using commercial TPA. We varied and tested the effects of bioconversion temperatures, reaction buffer compositions, tube size/aeration, and cell density on the conversion efficiency (Figure S25). We found that conversion temperatures (21 vs. 37°C), the presence of additives for PET degradation by MG8 (0.5 M NaCl and 5% DMSO), and the cell density had minimal effects on TPA conversion to catechol, as confirmed by HPLC. On the other hand, sufficient aeration seemed to have a strong effect on the bioconversion by CAT cells, with the use of a larger (15 mL) reaction tube size with better aeration giving tenfold higher conversion to catechol than a 1.5 mL tube (Figure S25). This is likely due to the requirement of the oxidative phase of the pentose phosphate pathway in regenerating NADPH for TPADO—a known pathway bottleneck [25] for the TPA‐to‐catechol conversion. We achieved ≈90% conversion of 10 mM TPA to catechol using CAT cells under an optimal condition (cells with OD_600_ ≈15 as input in a 15 mL tube at 37°C for 24 h).

We ultimately combined PET‐degrading cells secreting MG8^G127Y/F250A^ (PET cells) with CAT cells to produce an integrated cell‐based platform for the degradation of PET waste and valorization of resulting TPA to catechol (Figure S26). 9% crystalline postconsumer PET powder was first degraded with PET cells in the modified M9 media supplemented with 50 mM K_2_HPO_4_/KH_2_PO_4_ pH 8.0, 0.5 M NaCl and 5% v/v DMSO for 2 days at 37°C. Thereafter, CAT cells were added, and TPA upcycling to catechol allowed to proceed for 2–4 days. We found that providing 0.5% w/v glucose supplement to enhance NAD(P)H generation at the time of CAT cell addition to PET cells increased TPA‐to‐catechol conversion by 13%–50%, resulting in quantitative conversion of TPA to catechol (Figure S26). The initial PET degradation step could be extended to 4 and 7 days, where conversion of catechol from PET reached 3.7% and 7.8%, respectively (Figure 5b,c; Table S7).

To assess changes in strain ratio during co‐cultivation, we sampled PET‐CAT cells at 2, 4, and 7 days, performed serial dilutions, and plated on selective agar containing kanamycin (to which PET cells are resistant) or streptomycin (to which C cells are resistant). Over the 7‐day period, CAT cells progressively outcompeted PET cells (Figure S27 and Tables S8‐S9), likely due to reduced growth capacity of the latter following PET degradation. This change in strain ratio did not affect PET degradation or upcycling efficiency, as the two steps are carried out sequentially in the current protocol.

## 3 Discussion

In summary, we used structural and molecular dynamics information to guide targeted loop mutagenesis of the human saliva metagenomic PET hydrolase MG8 to improve its PET degradation activity, particularly for cell‐based applications. A G127Y/F250A double mutation and a N125S/G127Y/F250A triple mutation with activity enhancements were identified through a high‐throughput mass spectrometry screening platform. A secretion system was engineered in *E. coli* to express MG8^G127Y/F250A^, enabling efficient breakdown of PET. Finally, we combined “PET cells” with “CAT cells”, the latter engineered to convert TPA to catechol, creating PETCAT, a fully integrated microbial platform that degrades PET and transforms it into catechol, a valuable chemical feedstock.

A growing repertoire of PET hydrolases validated for in vitro depolymerization could be adapted for the cell‐based PET degradation platform, and different activity profiles of these enzymes in different buffers and media could offer complementary tools when they are used on diverse waste streams or impure feedstocks. Even with loop modifications presented here, our engineered MG8 is not as active as state‐of‐the‐art PET hydrolases such as engineered LCC, but there are specific cell‐compatible conditions with moderate‐to‐high NaCl concentrations in which MG8 works well to generate TPA from PET degradation at the same efficiency range as cell‐based PET degradation by PHL7, which has its own preference of high‐phosphate medium [43]. Further engineering and directed evolution of different PET hydrolases so they can withstand contaminants from different feedstocks under industry‐relevant conditions may be key in enabling biocatalytic recycling of diverse forms of PET in different controlled environments.

While microbial‐based degradation and recycling of PET plastics show promise, there are key limitations which require improvements before the platform can be useful. At the substrate level, mesophilic PET hydrolases including our engineered enzymes can likely only degrade low‐crystalline PET; degrading high‐crystallinity PET such as those found in consumer products still requires the development of cost‐effective and greener plastic pretreatment processes. Complementary pretreatment approaches which work across scales—from larger‐scale extrusion, cryo‐grinding and micronization [9], to microwave‐ [44] and solvent‐assisted [43] pretreatment compatible with smaller‐scale processes—are still needed. Future enzyme development should aim to create biocatalysts capable of withstanding impurities from diverse feedstocks and maintaining efficient degradation at high solid loadings. At the biochemical machinery level, one can take inspiration from how nature degrades crystalline polymers like cellulose and adapt multienzyme assembly approaches with auxiliary modules and enzymatic cascades to further promote PET degradation [45].

At the cellular level, the division of metabolic labor, as demonstrated by the PET and CAT strains, and concurrent development of strategies to stabilize and coordinate synthetic microbial consortia [46] are essential for the successful integration of multiple novel metabolic functions into microbial systems. Additional catabolic pathways—for example, for ethylene glycol (EG) [47]—and detoxification mechanisms to mitigate the effects of compounds such as excess TPA, can be incorporated into parallel strains. EG metabolism could be integrated to supply cofactors for the TPA‐to‐catechol bioconversion pathway, with an added caveat of increased metabolic burden from exogenous expression of required dehydrogenase enzymes. Ultimately, the efficient one‐pot upcycling of PET into higher‐value chemicals through metabolic engineering and biocompatible chemistry [26] can enhance the economic viability of using engineered living systems to capture and convert plastic waste into sustainable chemicals and materials.

## Supporting Information

Additional supporting information can be found online in the Supporting Information section. **Supporting Fig. S1:** a, SDS‐PAGE of MBP‐MG8 expression. b, Profile of SP cation exchange chromatography column to separate MG8 from MBP after TEV protease mediated cleavage. MG8 was eluted out at conductivity of 5 mS/cm whereas MBP was eluted out at the final step of the gradient of up to 150 mS/cm conductivity. **Supporting Fig. S2:** a, Numbering of secondary structure features of MG8. The structure comprised of 8 alpha‐helices and 9 beta‐strands. The catalytic triad residues and two disulfide bonds are highlighted in sticks. b, Similar to other mesophilic class II PET hydrolases, MG8 has two stabilizing disulfide bonds which were well‐resolved in the structure. c, Sequence alignment of MG8 and other mesophilic class II PET hydrolases, PE‐H, IsPETase, and Mors1. The secondary structure features were extracted from crystal structures and the key residues supporting PET degradation are highlighted. The cysteines forming stabilizing disulfide bonds are conserved in these enzymes. **Supporting Fig. S3** **:** Pooled sequencing results of MG8(NRT^71‐73^/F250A) and MG8(NRT^125‐127^/F250A) libraries, compared to their template sequences. **Supporting Fig. S4** **:** a, Expression of MG8, MBP‐tagged MG8, and LbCas12a (negative control) and their partitioning into supernatants of the cell lysates generated with BugBuster. MBP‐MG8 was largely soluble and could be extracted into lysates, while MG8 without MBP remained in the inclusion bodies. b, Ion suppression effects from cell lysates on TPA detection on RapidFire MS equipped with a C18 cartridge. We proceeded with using the lower amount (30 µL) of lysates as the signal to‐noise was better, and we could detect down to 0.01 µM TPA reliably. **Supporting Fig. S5** **:** Representative plots from the first heating cycle of PET‐film (top) and R‐PET film (bottom) for differential scanning calorimetric analyses. **Supporting Fig. S6** **:** MHET and TPA detected on the MRM mode of RapidFire MS at varying amount of BugBuster, and varying amount of lysates. Lysates were prepared from BL21(DE3) transformed with corresponding expression plasmids then induced for protein expression. The effect of cell lysate interference through monomer quantification was done using cell lysate of BL21. MHET was detected with the 209 to 121 MRM transitions, while TPA was detected with 165 to 121 transitions. **Supporting Fig. S7** **:** Multiple reaction monitoring (MRM) in a negative mode was used to characterize MHET and TPA products from PET degradation activity. MS1 ions for both MHET and TPA were used for quantification. **Supporting Fig. S8** **:** SDS‐PAGE of purified top 15 MG8 variants with an N‐terminal MBP tag. **Supporting Fig. S9** **:** Relative activity of eight purified MBP‐tagged MG8 variants from the b1‐b2 and b4‐ a3 loop libraries in hydrolyzing PET film to generate MHET (left) and TPA (right) (same underlying data as Figure 3a). Hydrolysis was performed for 2 days under different NaCl concentrations (0.01 ‐ 4 M) and temperatures (37°C and 55°C) before quenching with formic acid and assessed by HPLC. MBP‐tagged wild‐type MG8 and MG8^F250A^ act as reference points. Error bars, ± S.D. from triplicates. **Supporting Fig. S10** **:** Superior activities of MG8^G127Y/F250A^ and MG8^N125S/G127Y/F250A^ are maintained after MBP tag removal (same underlying data as Figure 3b). PET degradation reactions were set up as in Figure S8, then soluble PET degradation products assessed by absorbance measurements at 260 nm. MG8^S171A^ has its catalytic serine mutated and is mechanistically inactive. Error bars, ± S.D. from triplicates. **Supporting Fig. S11** **:** Thermal stabilities of MG8^G127Y/F250A^ and MG8^N125S/G127Y/F250A^ with MG8^F250A^ as a reference point. The effect of the MBP tag on T_m_ values of each MG8 variant as well as effects of NaCl concentrations on thermal stabilities are shown. **Supporting Fig. S12** **:** Backbone RMSF of MG8^G127Y/F250A^ and MG8^N125S/G127Y/F250A^ from the last 20 ns of 100 ns simulations at 37°C. Simulations for both enzymes were performed at 150 mM NaCl. The PET‐capturing loop, the catalytic aspartate loop and the catalytic histidine loop, all of which participate in reaction catalysis, are highlighted in purple. β1 – β2 and β4 – α3 loops are in orange. **Supporting Fig. S13** **:** Relative activity of MG8 variants in degrading different PET substrates at 37°C (same underlying data as Figure 3d). Assays were set up as in a with different PET substrates. The ICCM quadruple mutant of the leaf‐and‐branch compost cutinase (LCC) was used as an activity reference point. Error bars, ± S.D. from triplicates. **Supporting Fig. S14** **:** Relative activity of MG8^G127Y/F250A^, FAST‐PETase, and LCC^ICCM^ in hydrolyzing PET film (9% crystallinity) under 25 mM Glycine pH 9.0 and 4 M NaCl (blue graphs); or 100 mM phosphate buffer pH 8.0 (red graphs). PET degradation was performed for 2 days at 37°C. Error bars, ± S.D. from triplicates. **Supporting Fig. S15** **:** Effects of DMSO (a) and PET particle size (b) on enzymatic PET degradation by MG8. A_260_ measurements of soluble PET products from enzymatic PET degradation were collected from 48‐hour reactions at 37°C under 25 mM Glycine pH 9.0 with 1 M NaCl. a, MG8^G127Y/F250A^ was tested for depolymerization of PET film and particles and in the presence of 10% (v/v) DMSO. b, Time‐coursed measurements of degradation of different PET forms by MG8^G127Y/F250A^, as well as effects of 10% DMSO. Error bars, ± S.D. from triplicates. **Supporting Fig. S16** **:** Quantification of secreted MG8 variants. a, SDS‐PAGE analysis of secreted MG8^N125S/G127Y/F250A^ and MG8^G127Y/F250A^. The supernatants were collected from culture after expression at 25°C for 66 h. NC: negative control of E. coli BL21(DE3) without plasmid; MG8 size: 31.75 kDa. b, Protein concentration (μg/mL) was calculated from band intensity with a BSA standard curve at concentrations of 125, 62.5, 31.25, and 15.625 μg/mL. **Supporting Fig. S17** **:** Optimization of cell growth and secretion conditions for maximal secreted MG8 amount. a, Varying secretion times of 18, 24, 42, 48, and 66 h at 25°C and 1 mM IPTG. b, Varying IPTG concentrations (0.1, 0.25, 0.5, and 1 mM) at 25°C and 66 h secretion time. c, Varying culture temperatures of 18, 25, and 30°C during secretion with 1 mM IPTG and 48 h secretion time. d, Different media formulations: M9 medium supplemented with 0.5% w/v glucose, 1% v/v glycerol, and 0.5% w/v yeast extract; Luria broth, LB; and terrific broth, TB. The culture was induced at 25°C for 66 h with 1 mM IPTG. **Supporting Fig. S18** **:** a, Secreted MG8^G127Y/F250A^ could be easily purified via nickel affinity chromatography. b, Purity of secreted MG8^G127Y/F250A^ after purification. **Supporting Fig. S19** **:** Optimization of cell‐based PET degradation conditions. a, Effects of additives. BL21(DE3) was transformed with the expression plasmid for B1PelB‐MG8^G127Y/F250A^ and enzyme expression induced for 66 h at 25°C. 500 µL of the culture was then supplemented with different additives (0.5 M NaCl; 50 mM K_2_HPO_4_/KH_2_PO_4_ pH 8.0; 50 mM Tris pH 8.0 and 9.0; 50 mM glycine NaOH pH 9.0; 5‐10% v/v DMSO; and 2 mg/mL BSA) and used to hydrolyze 7 mg PET powder at 37°C for 2 days before quenching with PMSF. Generated MHET and TPA were then analyzed by HPLC. Error bars, ± S.D. from triplicates. b, Effects of combinations of additives. Cells and degradation of PET were prepared and analyses performed as in a. Error bars, ± S.D. from triplicates. Secreted MG8^G127Y/F250A^ concentrations before addition of PET powder for a and b were 56 and 88 μg/mL respectively. **Supporting Fig. S20** **:** Growth curves of BL21(DE3) in the presence of additives to promote PET degradation. Cultures were inoculated at 5% v/v in modified M9 supplemented with 50 μg/ml kanamycin. Thereafter, cells were supplemented with different additives of 0.5 M NaCl, 100 mM Tris, pH 8.0 or both additives. Cell growth was monitored for additional 28 h. **Supporting Fig. S21** **:** SDS‐PAGE analysis of secreted MG8^G127Y/F250A^ amount prior to the PET degradation reaction, and after 2 days of PET degradation, in the presence or absence of secretor cells. NC: negative control of E. coli BL21(DE3) without the MG8 expression plasmid. The “culture” contained secretor cells during PET degradation, while the “supernatant” condition only contained growth media with presecreted enzyme (but the secretor cells were removed by centrifugation). Triplicate results are shown for culture and supernatant conditions. MG8 size: 31.75 kDa. **Supporting Fig. S22** **:** a, SDS‐PAGE analysis of secreted MG8^G127Y/F250A^ concentrations over time. Duplicates are shown for each time point. b, Secreted protein concentrations over the course of PET degradation reaction time (2‐14 days). **Supporting Fig. S23** **:** Concentrations of secreted MG8^G127Y/F250A^ over time, with and without secretor cells. Enzymes were first expressed and secreted for 66 h at 25°C before the PET degradation reactions at 37°C for 1, 2, 3, 4, and 7 days. Error bars, ± S.D. from triplicates. **Supporting Fig. S24** **:** Representative SDS‐PAGE analyses to assess concentrations of secreted MG8^G127Y/F250A^ over time, with and without secretor cells. (same underlying data as Figure S23). a, MG8^G127Y/F250A^ after secretion at 25°C for 66 h. b‐f, secreted MG8^G127Y/F250A^ after PET‐degrading reactions at 37°C for 1, 2, 3, 4, and 7 days, respectively. **Supporting Fig. S25** **:** Optimization of catechol bioconversion conditions. Effects of culture temperature, cell density, and tube size/aeration on catechol bioconversion were assessed. The reactions were performed with 10 mM standard TPA in 1.5 mL tube or 15 mL tube, and modified M9 (0.5% w/v glucose, 1% v/v glycerol, and 0.5% w/v yeast extract) supplemented 0.5 M NaCl, 50 mM K_2_HPO_4_/KH_2_PO_4_ pH 8.0, and 5% v/v DMSO at 21 and 37°C for 21 h. Error bars, ± S.D. from triplicates. **Supporting Fig. S26** **:** Effect of supplemented glucose to TPA‐to‐catechol conversion yields. a, Generated MHET and TPA from PET degradation prior to CAT‐cell addition (0 day) and after 2‐4 days of incubation with CAT cells, with and without 0.5% glucose supplementation. b, Catechol bioconversion from produced TPA after CAT‐cell addition for 2‐4 days. Generated catechol concentrations (mM, left) and percent conversion to catechol relative to initial TPA amount from cell‐based PET degradation (right) are shown. Error bars, ± S.D. from triplicates. Secreted MG8^G127Y/F250A^ concentrations before supplementation of PET powder: 69 μg/mL. **Supporting Fig. S27** **:** Assessing the presence of PET and CAT cells upon co‐culturing over time. PET cells were first used to degrade PET powder at 37°C for 2, 4, and 7 days. Thereafter, CAT cells were added to convert produced TPA to catechol at 37°C for 2 days. The mixed cultures from before and after the 2‐day reaction with CAT cells were serially diluted to final dilutions ranging from 10 ^4^to 10^‐7^ and spread on LB agar supplemented with 50 μg/mL kanamycin (of which PET cells are resistant) and 100 μg/mL streptomycin (of which CAT cells are resistant). The plates were incubated at 37°C for 16‐18 h before colonies were counted. +Kan: PET‐cell growth was performed in the presence of 12.5 μg/mL kanamycin. ‐Kan: PET‐cell growth was performed in the absence of kanamycin. **Supporting** **Table** **S1:** Data collection and refinement statistics of MG8 (PDB 9VZR). **Supporting Table S2:** Primers sequence. **Supporting** **Table** **S3:** Crystallinity of PET substrates. **Supporting Table S4:** Normalized PET degradation activities of 30 best variants from the MG8(NRT^71 73^/F250A) library. **Supporting Table S5** **:** Normalized PET degradation activities of 30 best variants from the MG8(NRT^125 127^/F250A) library. **Supporting Table S6** **:** Comparison of percent depolymerization for PET cell‐based degradation. **Supporting Table S7** **:** Comparison of percent conversion from PET for cell‐based valorization. **Supporting Table S8** **:** Number of colonies of PET and CAT cells after the sequential PET cell‐based degradation of PET (2, 4, or 7 days) and CAT cell‐based conversion of TPA to catechol. **Supporting Table S9** **:** Number of colonies of PET cells—which were grown with and without supplementation of 12.5 μg/mL kanamycin during the first PET degradation step—after co culturing with CAT cells for 2 days.

## Funding

This work is supported by National Science Research and Innovation Fund (NSRF) via the Program Management Unit for Human Resources & Institutional Development, Research and Innovation (B42G670039); UK Research and Innovation (MR/S033882/1); Biotechnology and Biological Sciences Research Council (BB/Y007972/1).

## Conflicts of Interest

C. U., P. M., R. A., and B. E. have filed a patent on engineered MG8 with improved activities. All other authors declare no competing interests.

## Supporting information

Supplementary Material

## Data Availability

The crystal structure of MG8 can be found under the PDB accession code 9VZR. All datasets generated and analyzed here are available from the corresponding authors upon reasonable request.
