# Supplementary material for: Cellular Upcycling of Polyethylene Terephthalate (PET) With an Engineered Human Saliva Metagenomic PET Hydrolase
Source: ChemSusChem. 2025 Dec 8;19(1):e202502560. doi: 10.1002/cssc.202502560 (PMC12767563; doi:10.1002/cssc.202502560)
Supplement: Supplementary file 1 — Supplementary Material [file CSSC-19-e202502560-s001.pdf]

Supporting Information

Cellular Upcycling of Polyethylene Terephthalate (PET) with an Engineered Human Saliva Metagenomic PET Hydrolase

Rawiporn Amornloetwattana[1,#], Bhumrapee Eiamthong[1,#], Piyachat Meesawat[1,#], Piyakamon Bunkum[1], Benjamin Royer[2], Nicoll Zeballos[2], Marcos Valenzuela-Ortega[2], Robert C. Robinson[1], Stephen Wallace[2,*], Chayasith Uttamapinant[1,*]

[1]School of Biomolecular Science and Engineering, Vidyasirimedhi Institute of Science and Technology (VISTEC), Rayong 21210, Thailand

[2]Institute of Quantitative Biology, Biochemistry and Biotechnology, School of Biological Sciences, University of Edinburgh, Edinburgh EH9 3FF, United Kingdom

[#]These authors contribute equally to this work

*Correspondence: chayasith.u@vistec.ac.th, Stephen.wallace@ed.ac.uk

The supporting information file contains Figures S1-S27, Table S1-S9,

and Materials and Methods

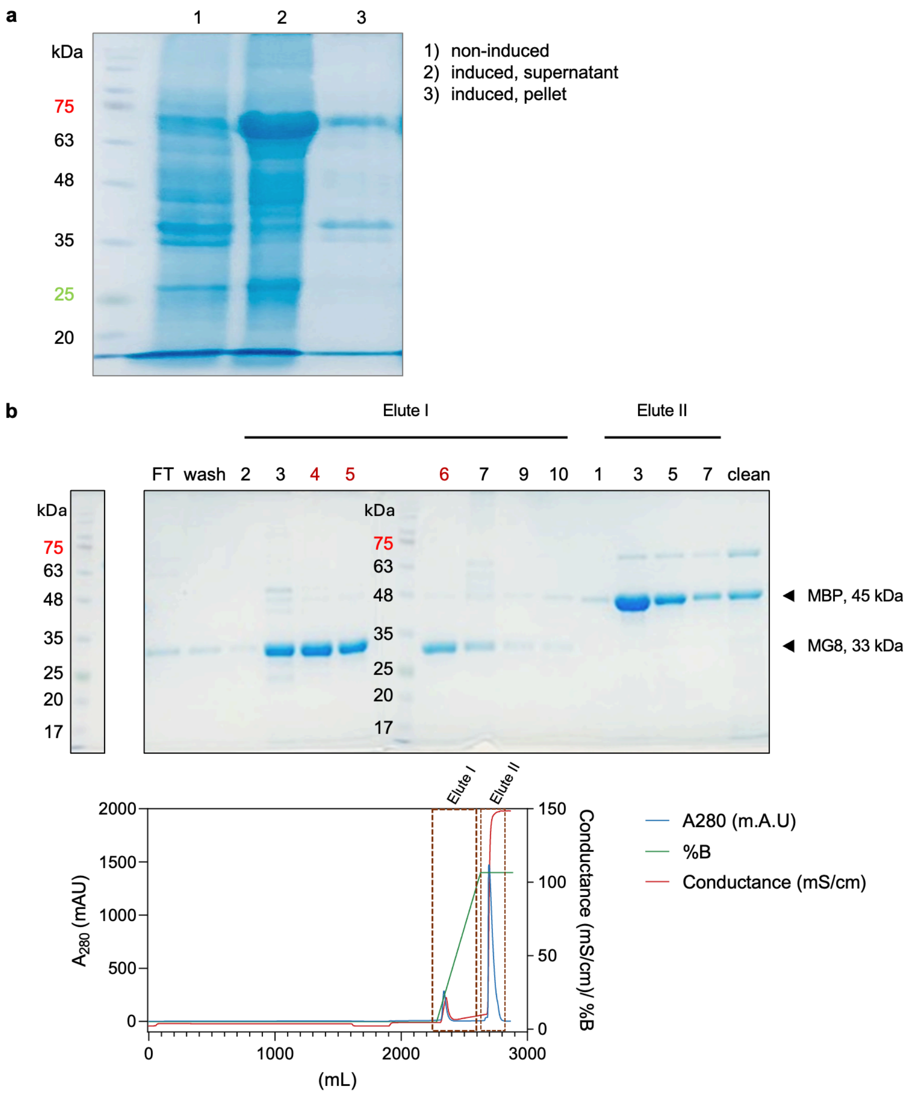

Figure S1. **a**, SDS-PAGE of MBP-MG8 expression. **b**, Profile of SP cation exchange chromatography column to separate MG8 from MBP after TEV protease-mediated cleavage. MG8 was eluted out at conductivity of 5 mS/cm whereas MBP was eluted out at the final step of the gradient of up to 150 mS/cm conductivity.

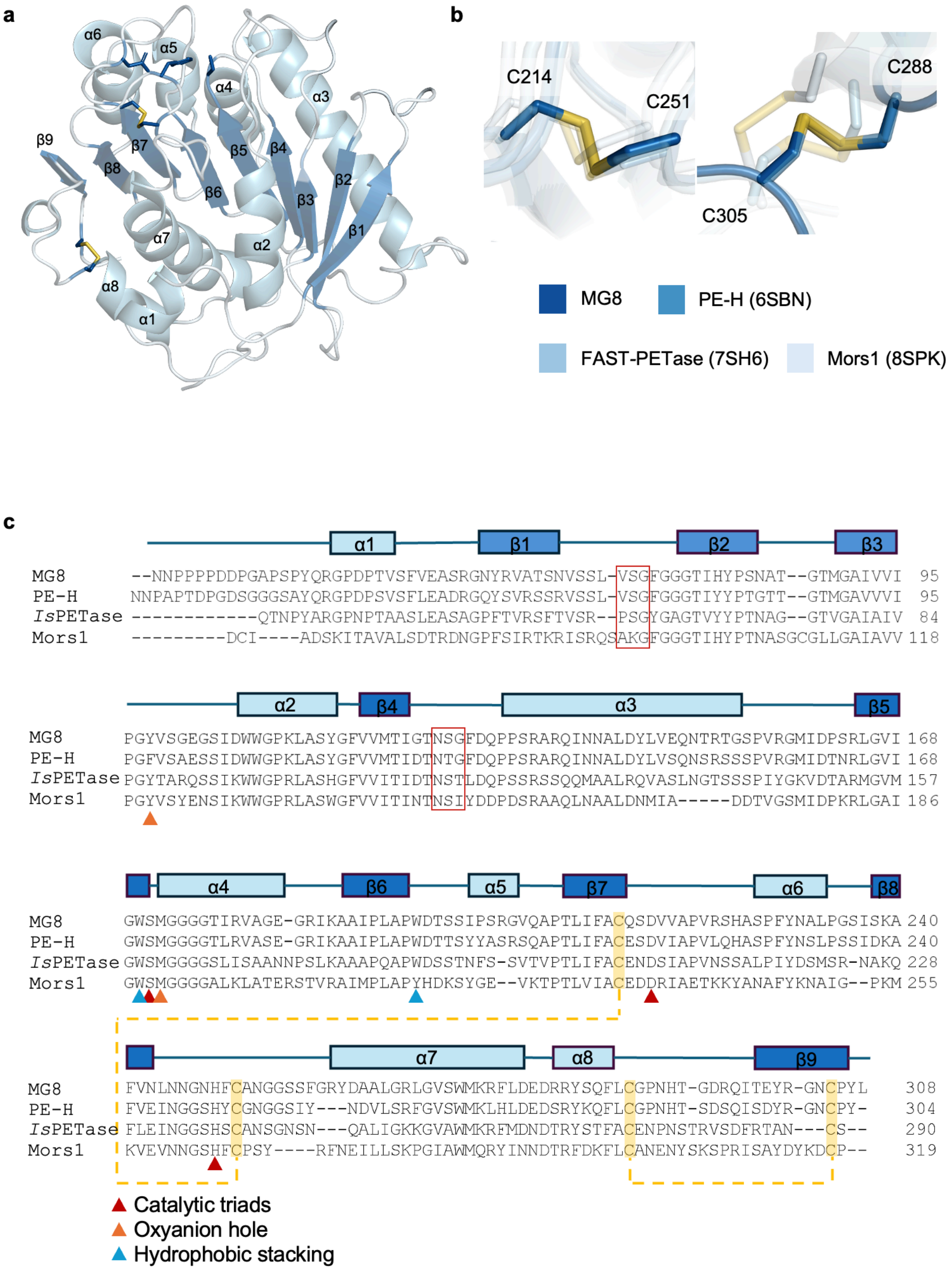

Figure S2. a, Numbering of secondary structure features of MG8. The structure comprised of 8 alpha-helices and 9 beta-strands. The catalytic triad residues and two disulfide bonds are highlighted in sticks. **b**, Similar to other mesophilic class II PET hydrolases, MG8 has two stabilizing disulfide bonds which were well-resolved in the structure. **c**, Sequence alignment of MG8 and other mesophilic class II PET hydrolases, PE-H, *Is*PETase, and Mors1. The secondary structure features were extracted from crystal structures and the key residues supporting PET degradation are highlighted. The cysteines forming stabilizing disulfide bonds are conserved in these enzymes.

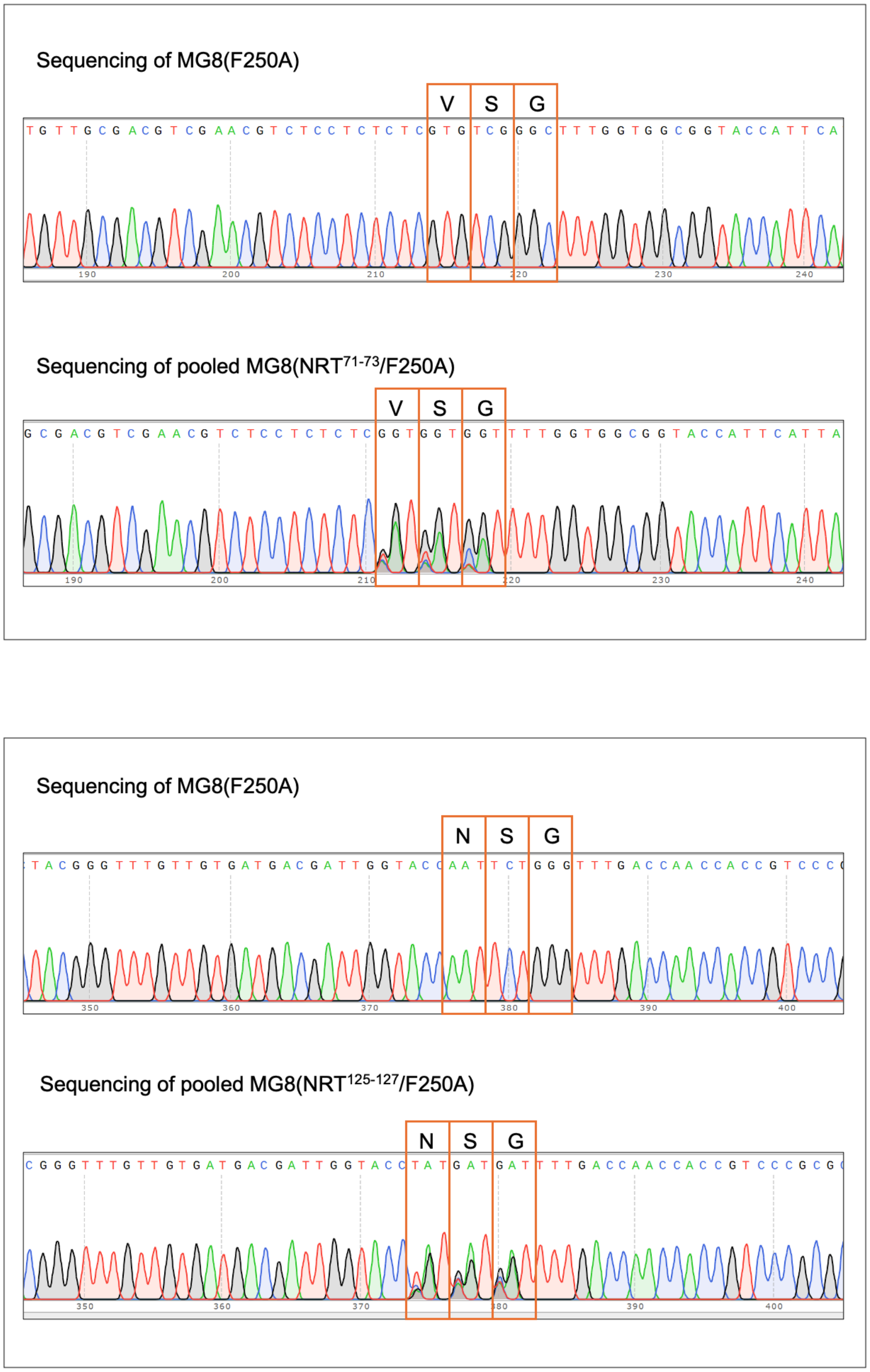

Figure S3. Pooled sequencing results of MG8(NRT$^{71\text{-}73}$/F250A) and MG8(NRT$^{125\text{-}127}$/F250A) libraries, compared to their template sequences.

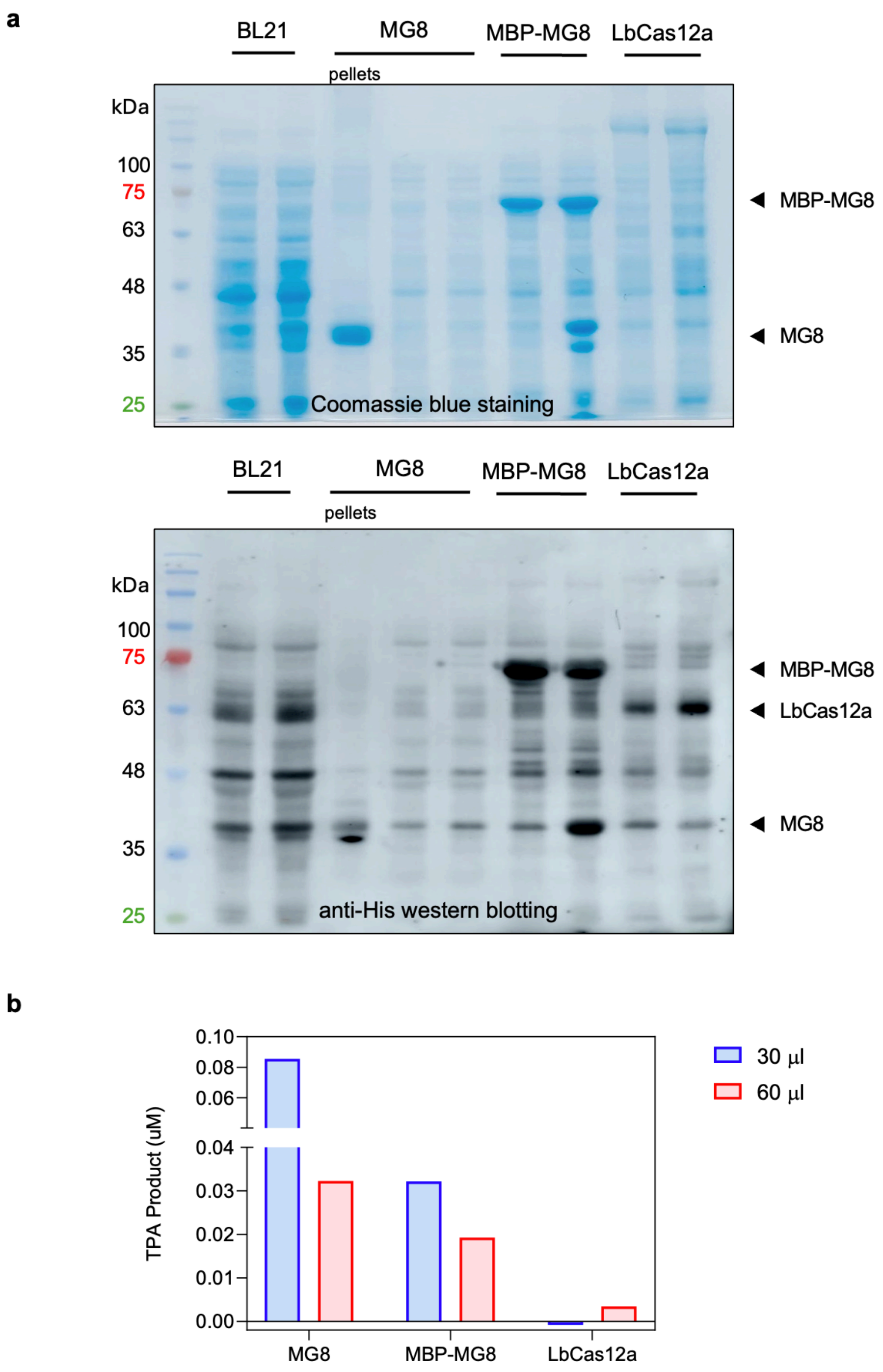

Figure S4. **a**, Expression of MG8, MBP-tagged MG8, and LbCas12a (negative control) and their partitioning into supernatants of the cell lysates generated with BugBuster. MBP-MG8 was largely soluble and could be extracted into lysates, while MG8 without MBP remained in the inclusion bodies. **b**, Ion suppression effects from cell lysates on TPA detection on RapidFire MS equipped with a C18 cartridge. We proceeded with using the lower amount (30 µL) of lysates as the signal-to-noise was better, and we could detect down to 0.01 µM TPA reliably.

Representative DSC results of PET film 6 mm

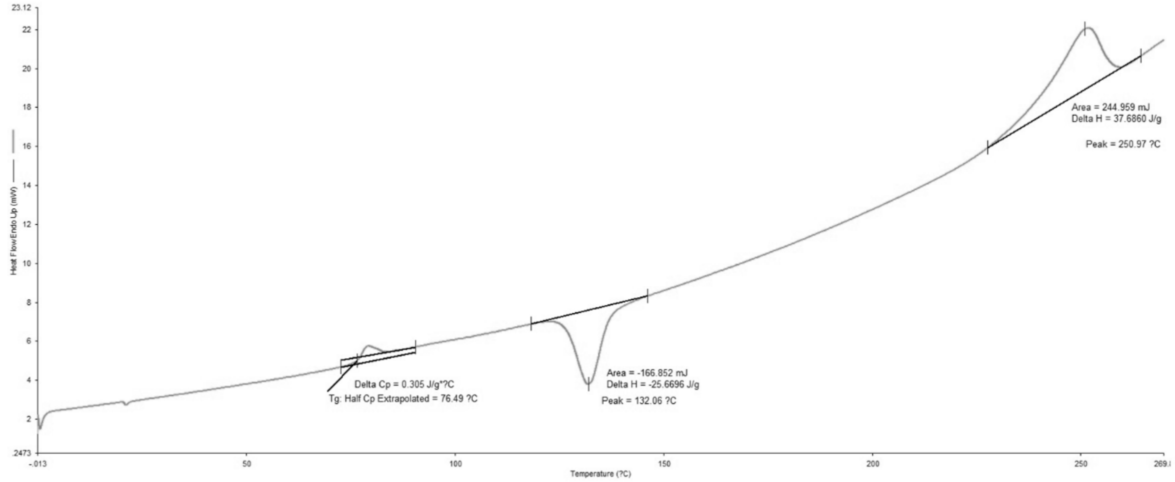

Representative DSC results of R-PET film 6 mm

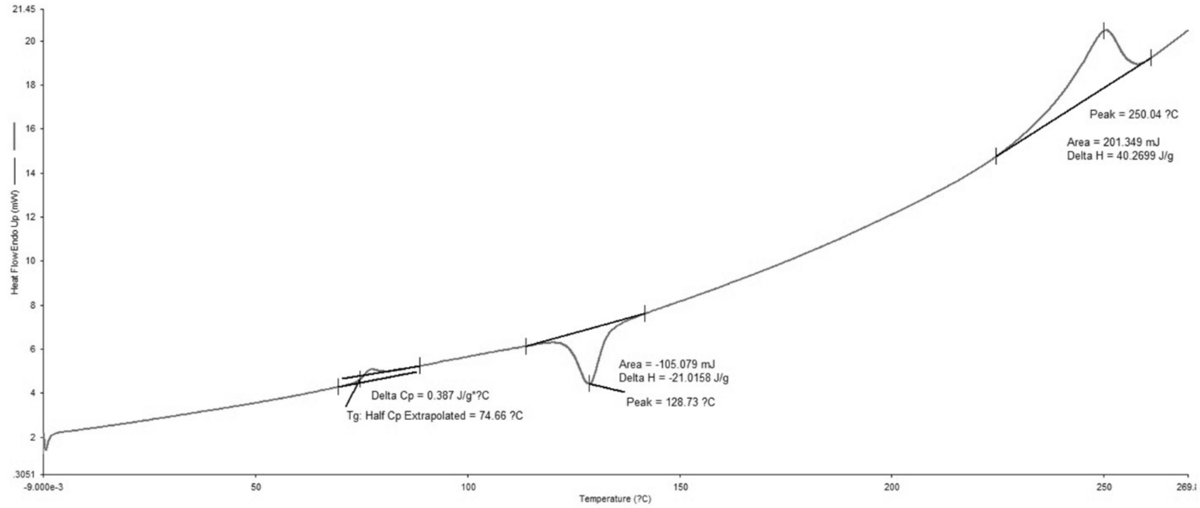

Figure S5. Representative plots from the first heating cycle of PET-film (top) and R-PET film (bottom) for differential scanning calorimetric analyses.

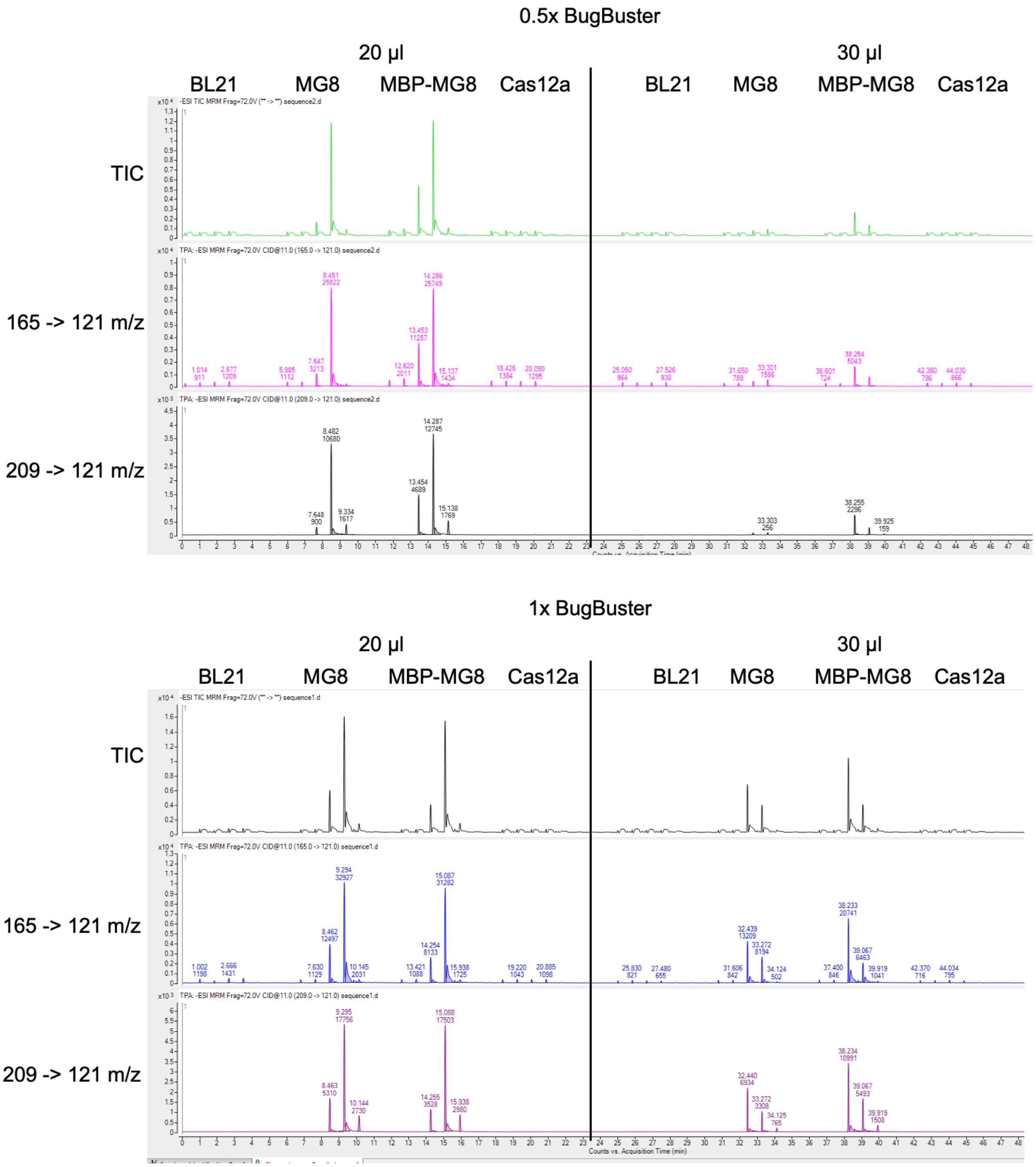

Figure S6. MHET and TPA detected on the MRM mode of RapidFire MS at varying amount of BugBuster, and varying amount of lysates. Lysates were prepared from BL21(DE3) transformed with corresponding expression plasmids then induced for protein expression. The effect of cell lysate interference through monomer quantification was done using cell lysate of BL21. MHET was detected with the 209 to 121 MRM transitions, while TPA was detected with 165 to 121 transitions.

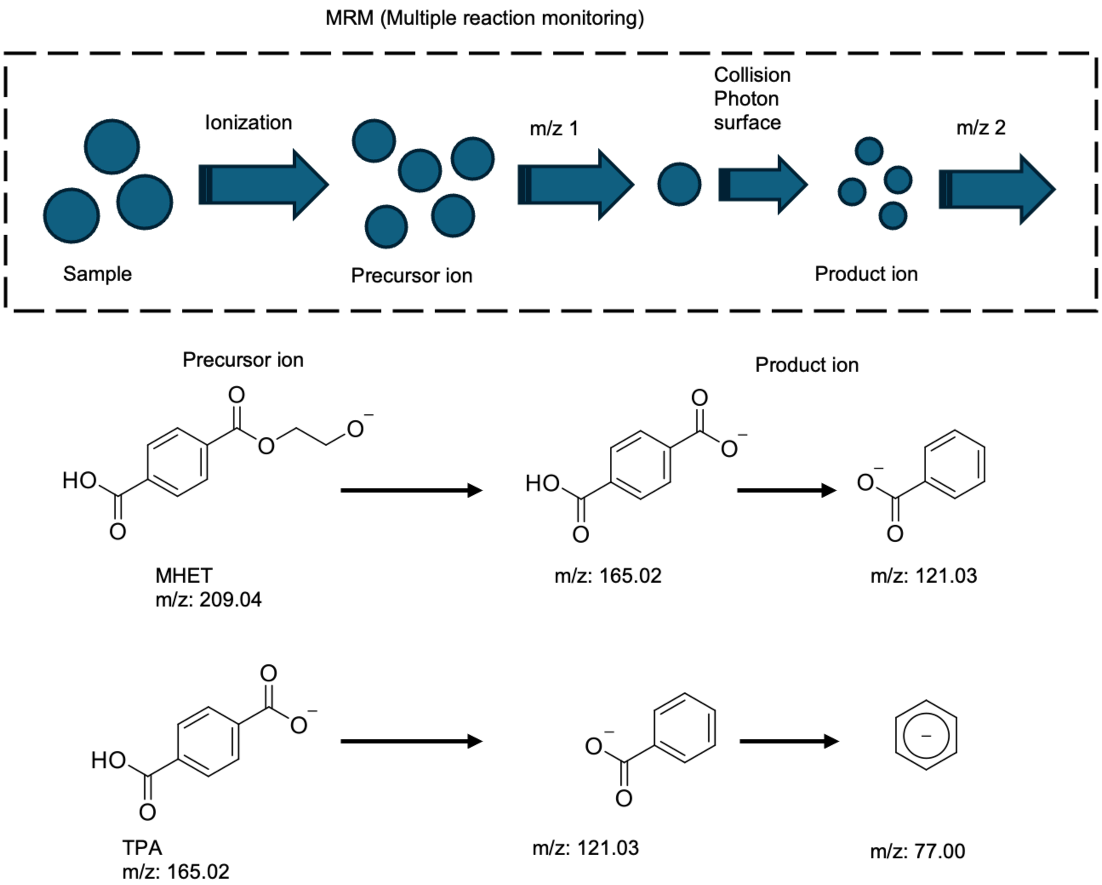

Figure S7. Multiple reaction monitoring (MRM) in a negative mode was used to characterize MHET and TPA products from PET degradation activity. MS1 ions for both MHET and TPA were used for quantification.

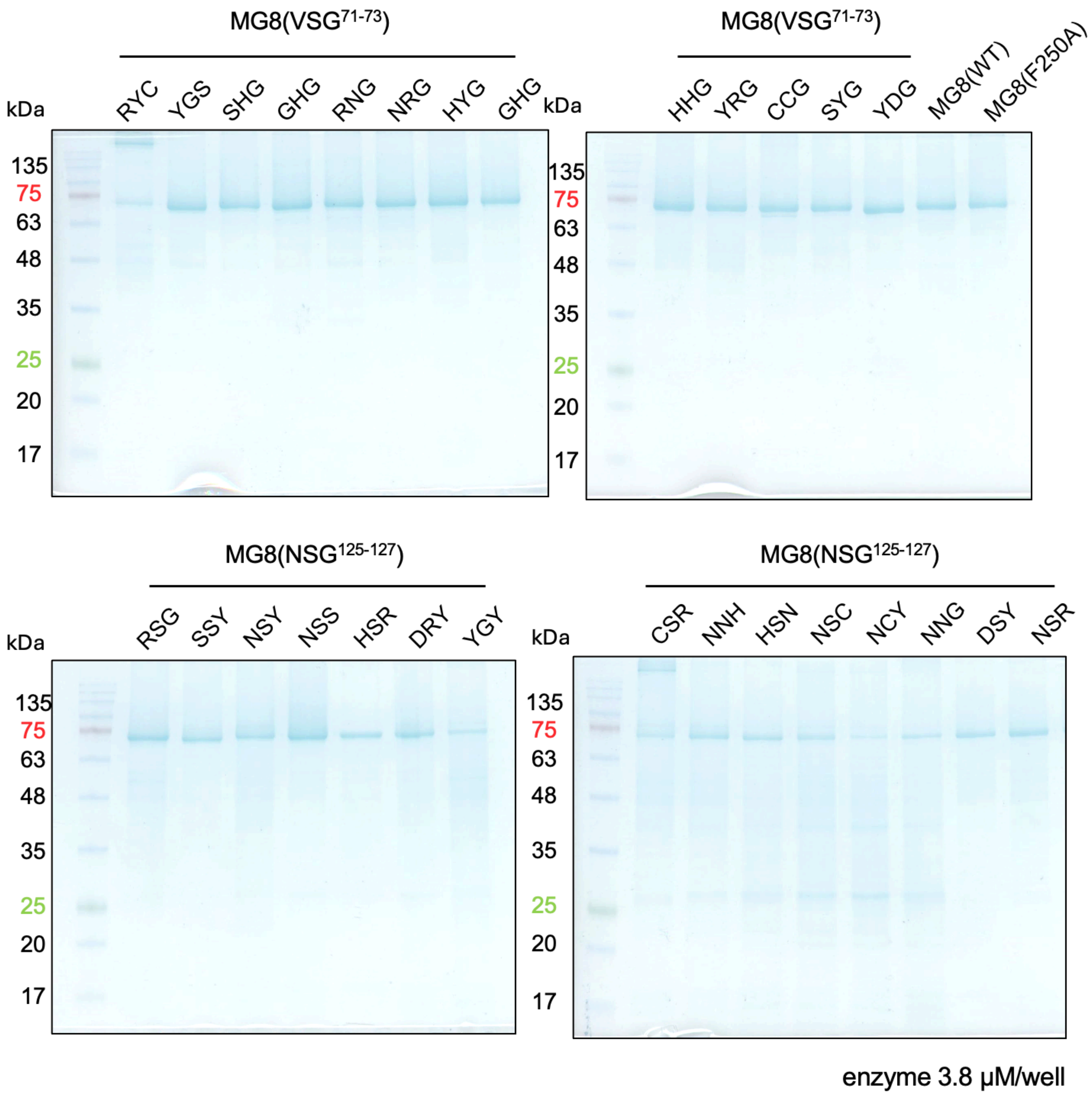

Figure S8. SDS-PAGE of purified top 15 MG8 variants with an N-terminal MBP tag.

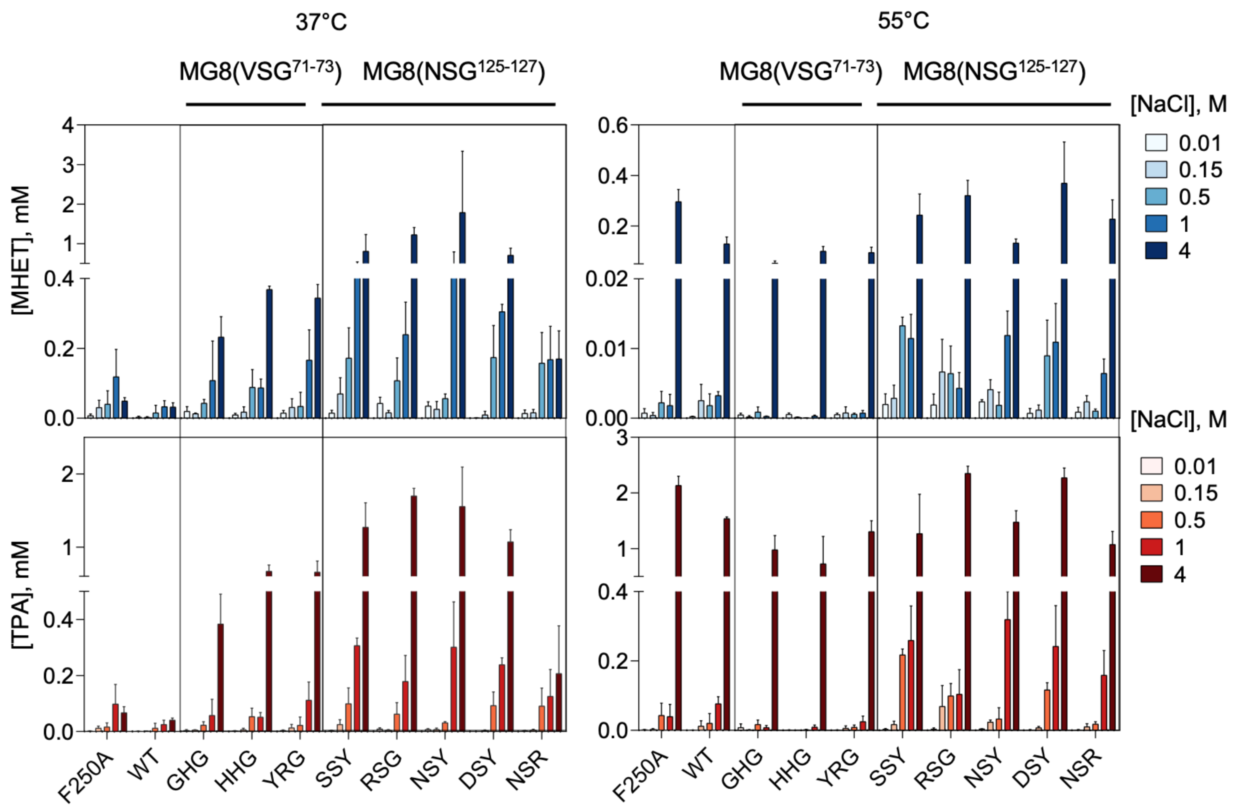

Figure S9. Relative activity of eight purified MBP-tagged MG8 variants from the β1–β2 and β4–α3 loop libraries in hydrolyzing PET film to generate MHET (left) and TPA (right) (same underlying data as Figure 3a). Hydrolysis was performed for 2 days under different NaCl concentrations (0.01 - 4 M) and temperatures (37 °C and 55 °C) before quenching with formic acid and assessed by HPLC. MBP-tagged wild-type MG8 and MG8^{F250A} act as reference points. Error bars, ± S.D. from triplicates.

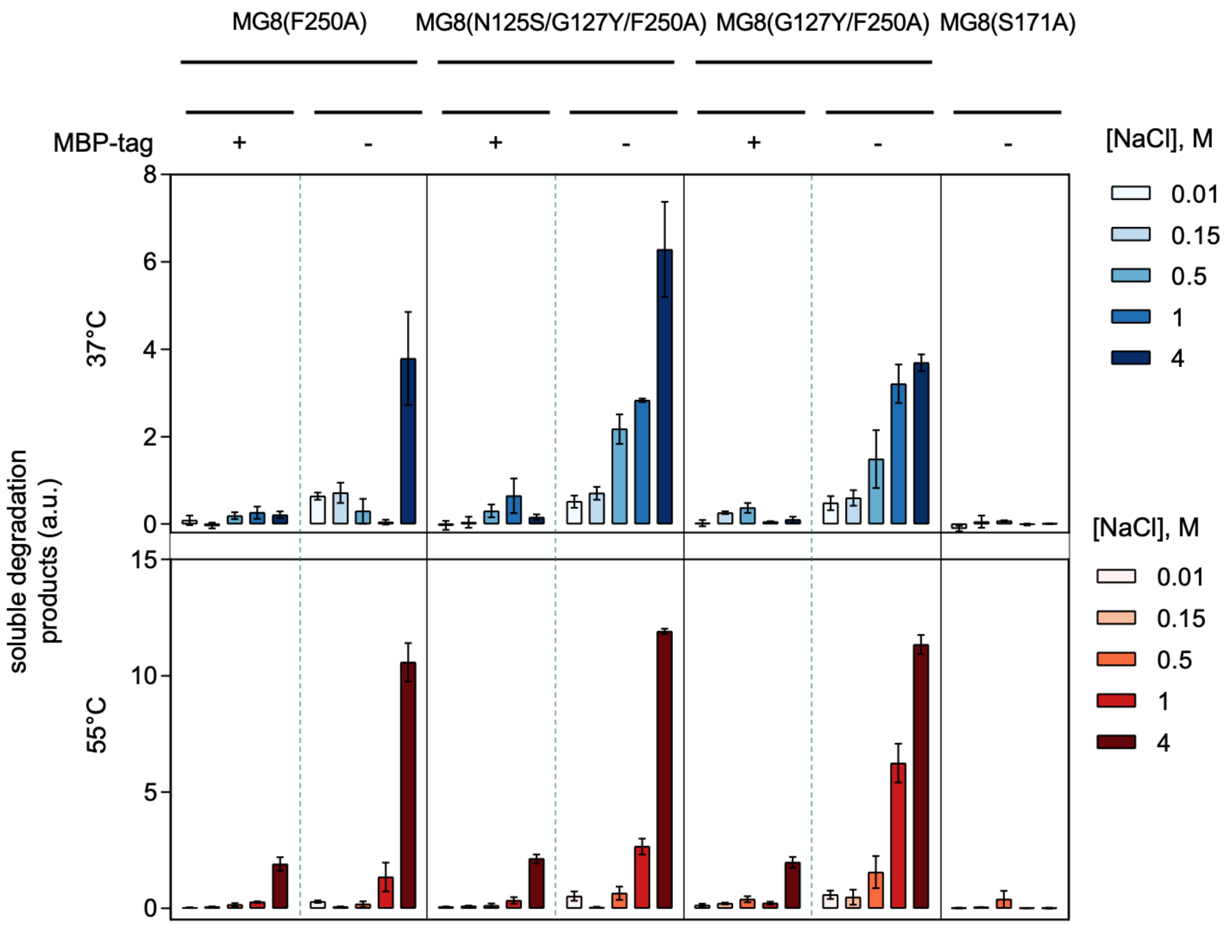

Figure S10. Superior activities of MG8$^{G127Y/F250A}$ and MG8$^{N125S/G127Y/F250A}$ are maintained after MBP tag removal (same underlying data as Figure 3b). PET degradation reactions were set up as in Figure S8, then soluble PET degradation products assessed by absorbance measurements at 260 nm. MG8^{S171A} has its catalytic serine mutated and is mechanistically inactive. Error bars, ± S.D. from triplicates.

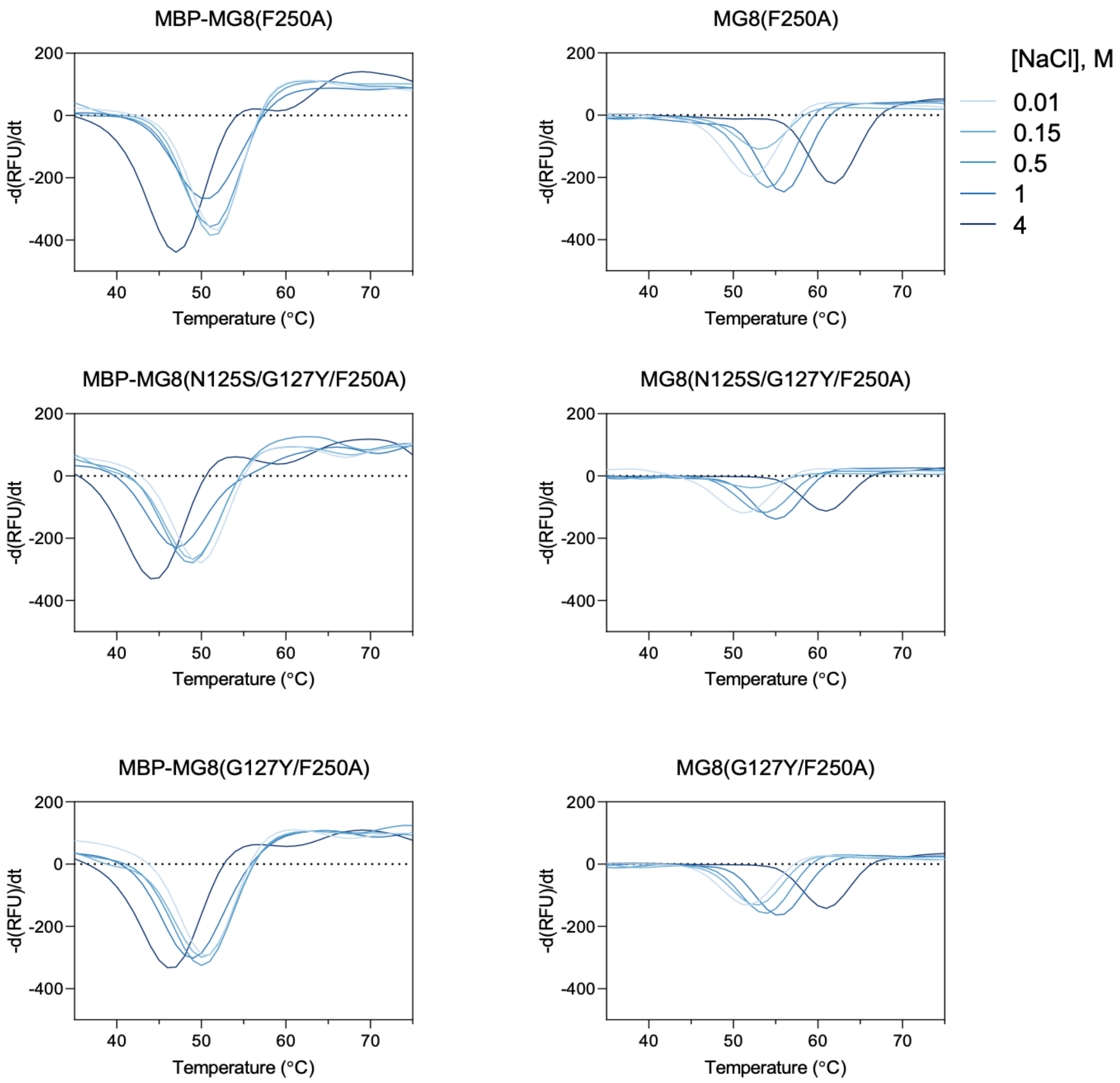

Figure S11. Thermal stabilities of $MG8^{G127Y/F250A}$ and $MG8^{N125S/G127Y/F250A}$ with $MG8^{F250A}$ as a reference point. The effect of the MBP tag on T_m values of each MG8 variant as well as effects of NaCl concentrations on thermal stabilities are shown.

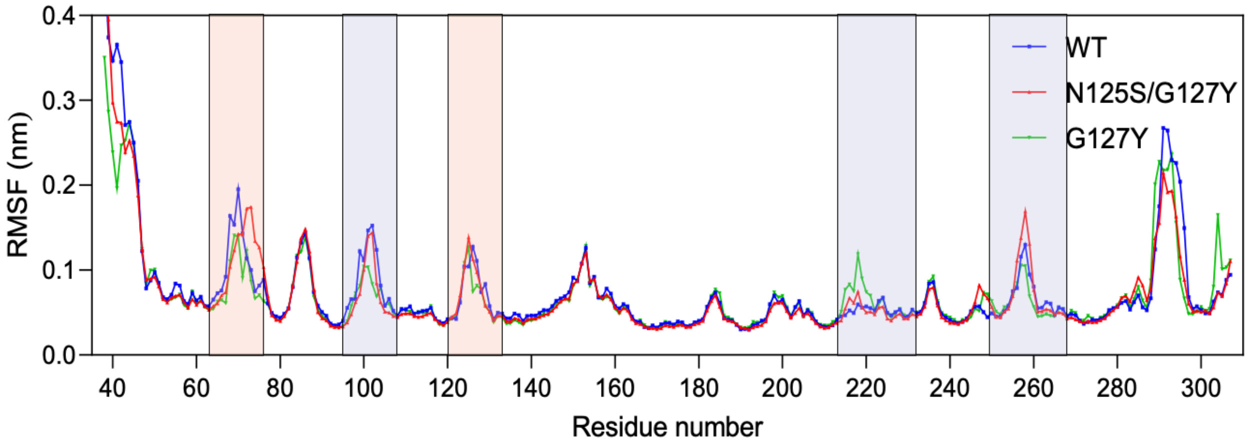

Figure S12. Backbone RMSF of $MG8^{G127Y/F250A}$ and $MG8^{N125S/G127Y/F250A}$ from the last 20 ns of 100 ns simulations at 37 °C. Simulations for both enzymes were performed at 150 mM NaCl. The PET-capturing loop, the catalytic aspartate loop and the catalytic histidine loop, all of which participate in reaction catalysis, are highlighted in purple. β1 – β2 and β4 – α3 loops are in orange.

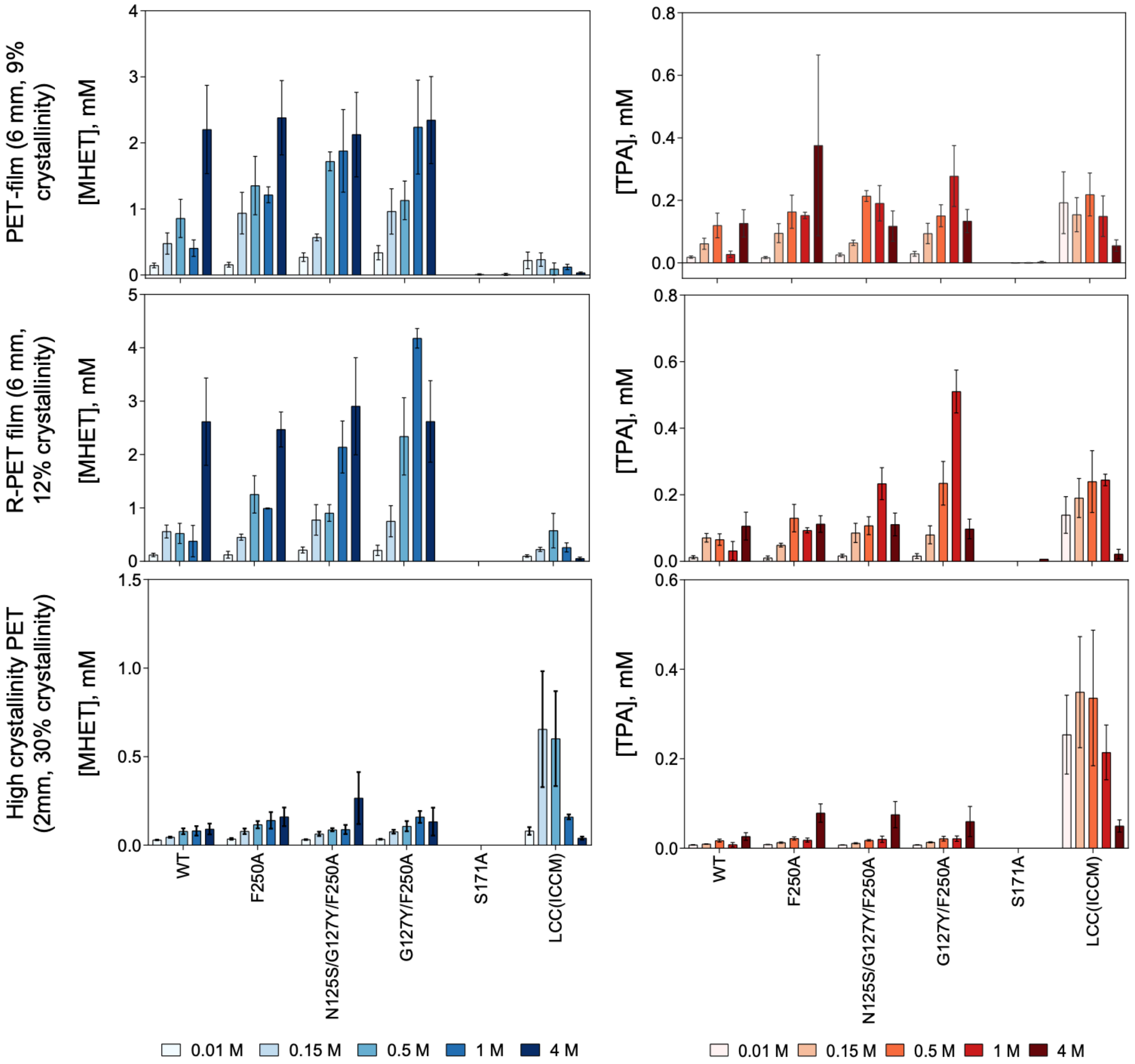

Figure S13. Relative activity of MG8 variants in degrading different PET substrates at 37°C (same underlying data as Figure 3d). Assays were set up as in **a** with different PET substrates. The ICCM quadruple mutant of the leaf-and-branch compost cutinase (LCC) was used as an activity reference point. Error bars, ± S.D. from triplicates.

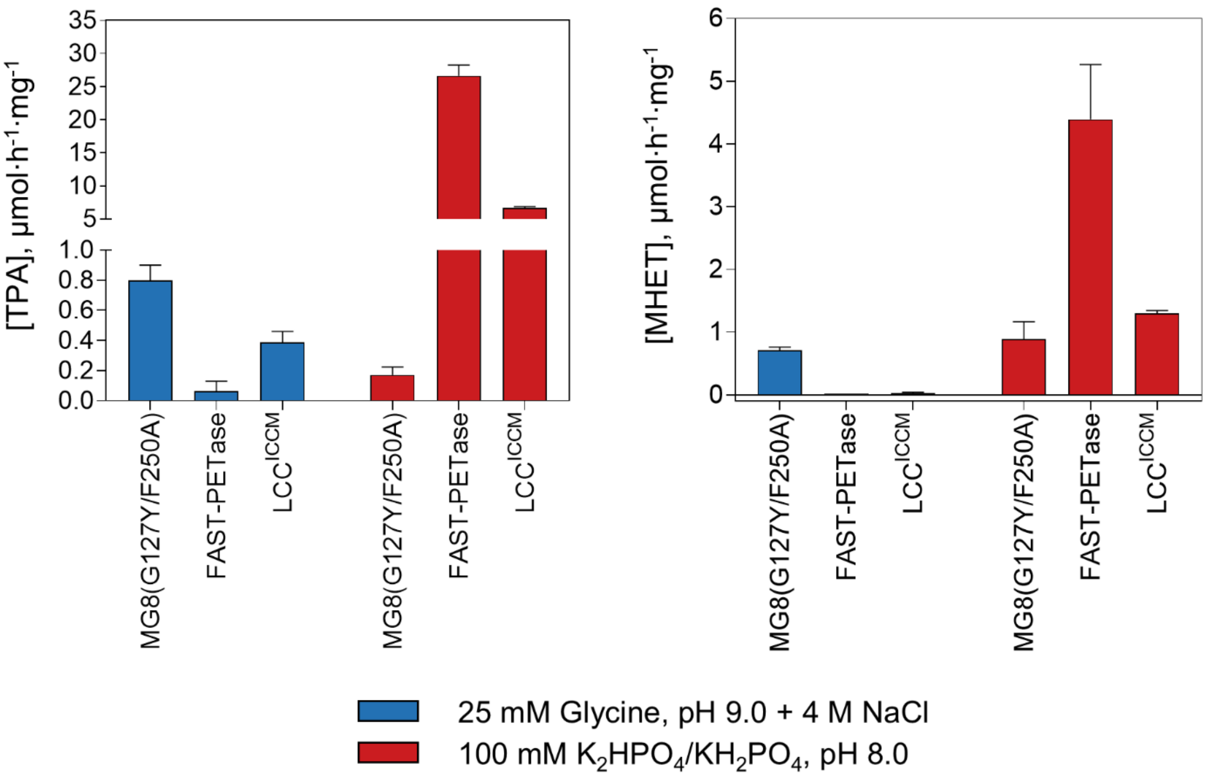

Figure S14. Relative activity of MG8$^{G127Y/F250A}$, FAST-PETase, and LCC^{ICCM} in hydrolyzing PET film (9% crystallinity) under 25 mM Glycine pH 9.0 and 4 M NaCl (blue graphs); or 100 mM phosphate buffer pH 8.0 (red graphs). PET degradation was performed for 2 days at 37 °C. Error bars, ± S.D. from triplicates.

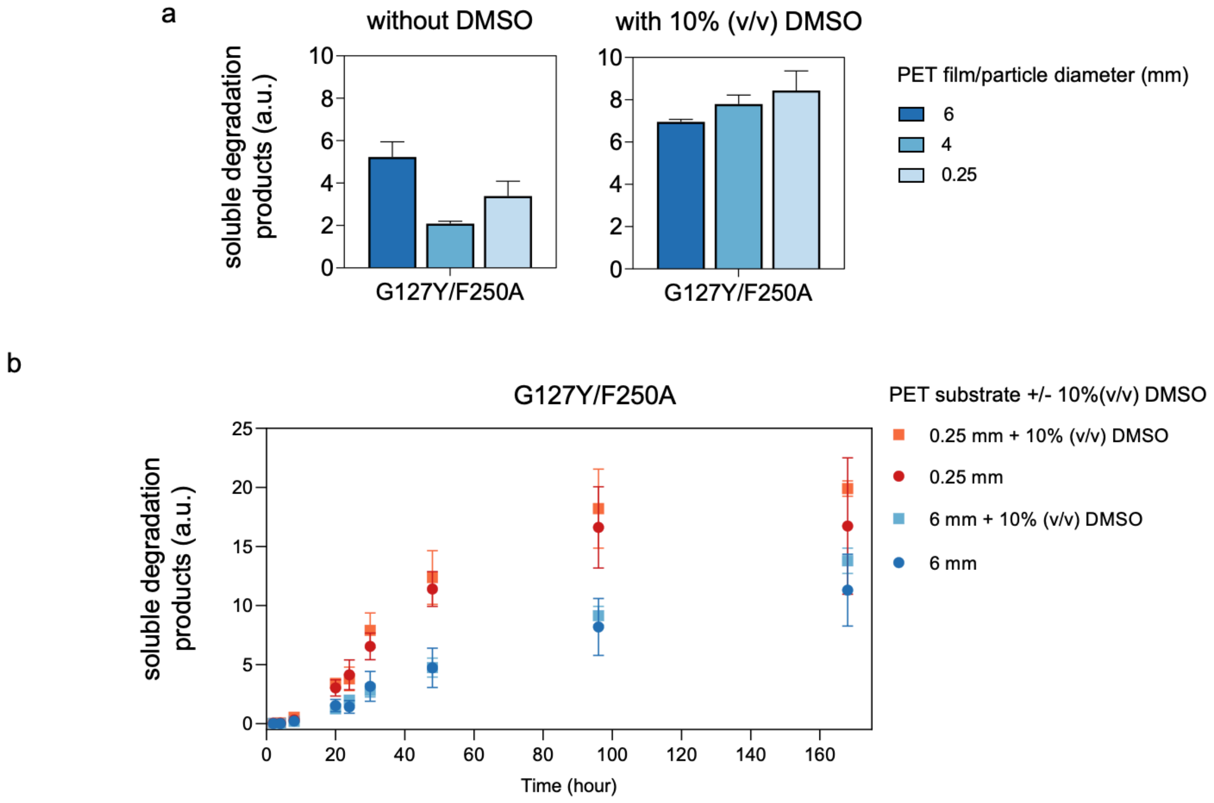

Figure S15. Effects of DMSO (**a**) and PET particle size (**b**) on enzymatic PET degradation by MG8. A_{260} measurements of soluble PET products from enzymatic PET degradation were collected from 48-hour reactions at 37 °C under 25 mM Glycine pH 9.0 with 1 M NaCl. **a**, $MG8^{G127Y/F250A}$ was tested for depolymerization of PET film and particles and in the presence of 10%(v/v) DMSO. **b**, Time-coursed measurements of degradation of different PET forms by $MG8^{G127Y/F250A}$, as well as effects of 10% DMSO. Error bars, ± S.D. from triplicates.

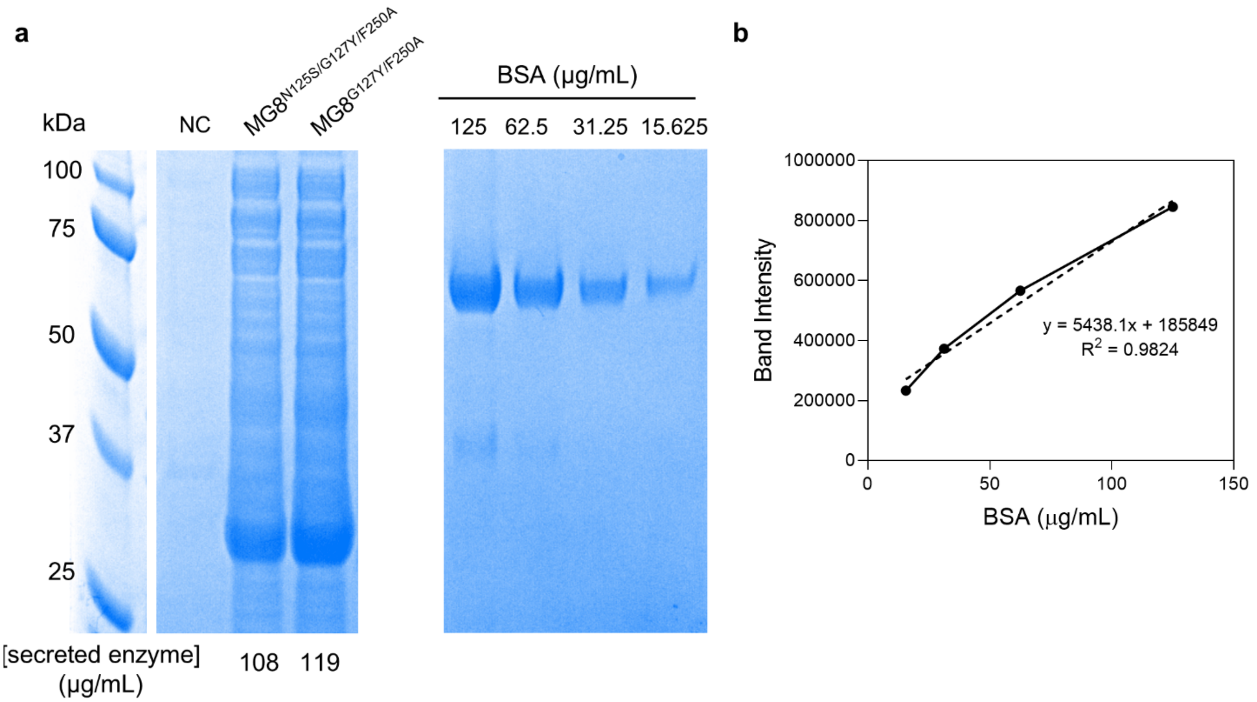

Figure S16. Quantification of secreted MG8 variants. **a**, SDS-PAGE analysis of secreted MG8 $^{N125S/G127Y/F250A}$ and MG8$^{G127Y/F250A}$. The supernatants were collected from culture after expression at 25 °C for 66 h. NC: negative control of *E. coli* BL21(DE3) without plasmid; MG8 size: 31.75 kDa. **b**, Protein concentration (μg/mL) was calculated from band intensity with a BSA standard curve at concentrations of 125, 62.5, 31.25, and 15.625 μg/mL.

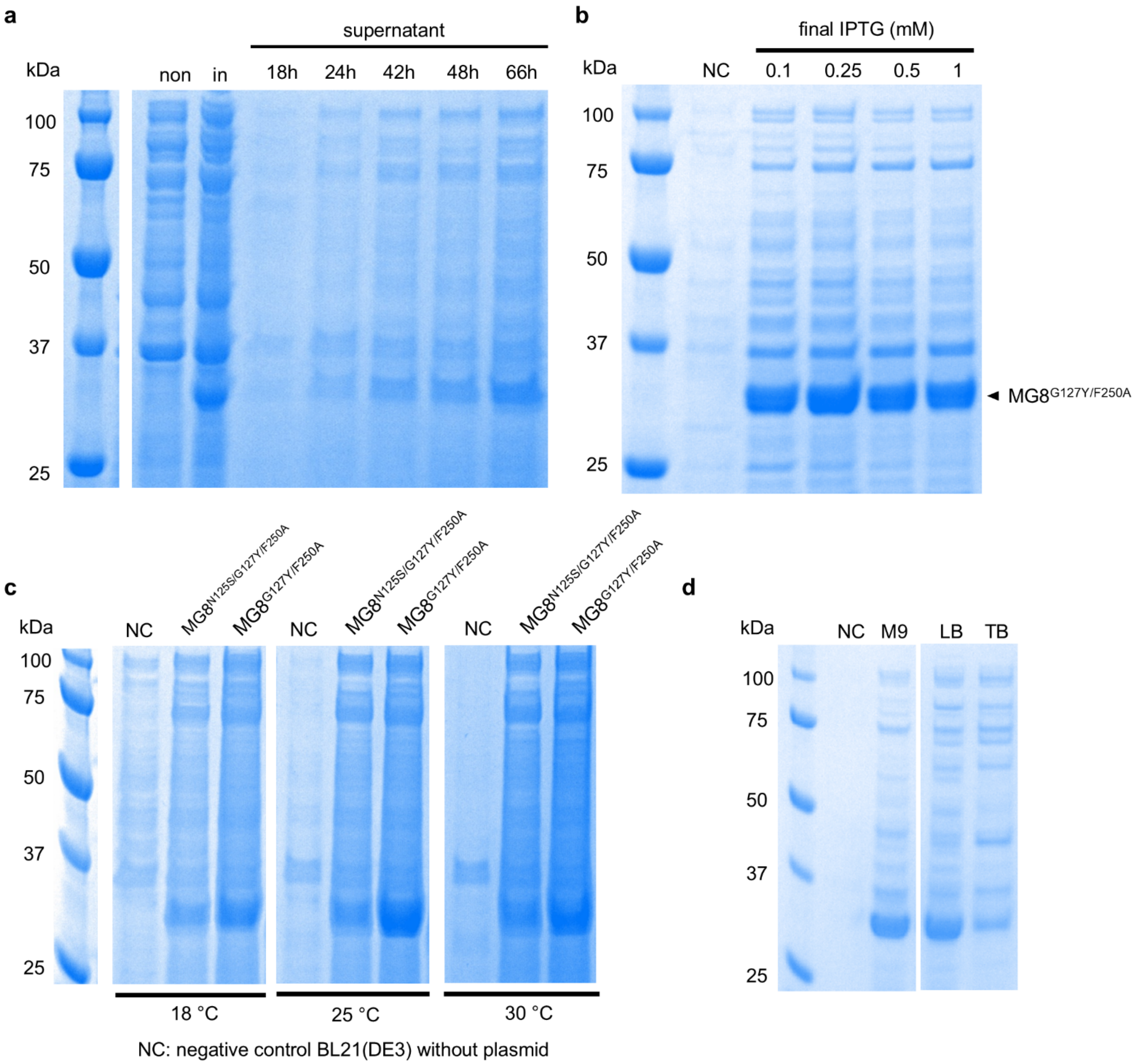

Figure S17. Optimization of cell growth and secretion conditions for maximal secreted MG8 amount. **a**, Varying secretion times of 18, 24, 42, 48, and 66 h at 25 °C and 1 mM IPTG. **b**, Varying IPTG concentrations (0.1, 0.25, 0.5, and 1 mM) at 25 °C and 66 h secretion time. **c**, Varying culture temperatures of 18, 25, and 30 °C during secretion with 1 mM IPTG and 48 h secretion time. **d**, Different media formulations: M9 medium supplemented with 0.5% w/v glucose, 1% v/v glycerol, and 0.5% w/v yeast extract; Luria broth, LB; and terrific broth, TB. The culture was induced at 25 °C for 66 h with 1 mM IPTG.

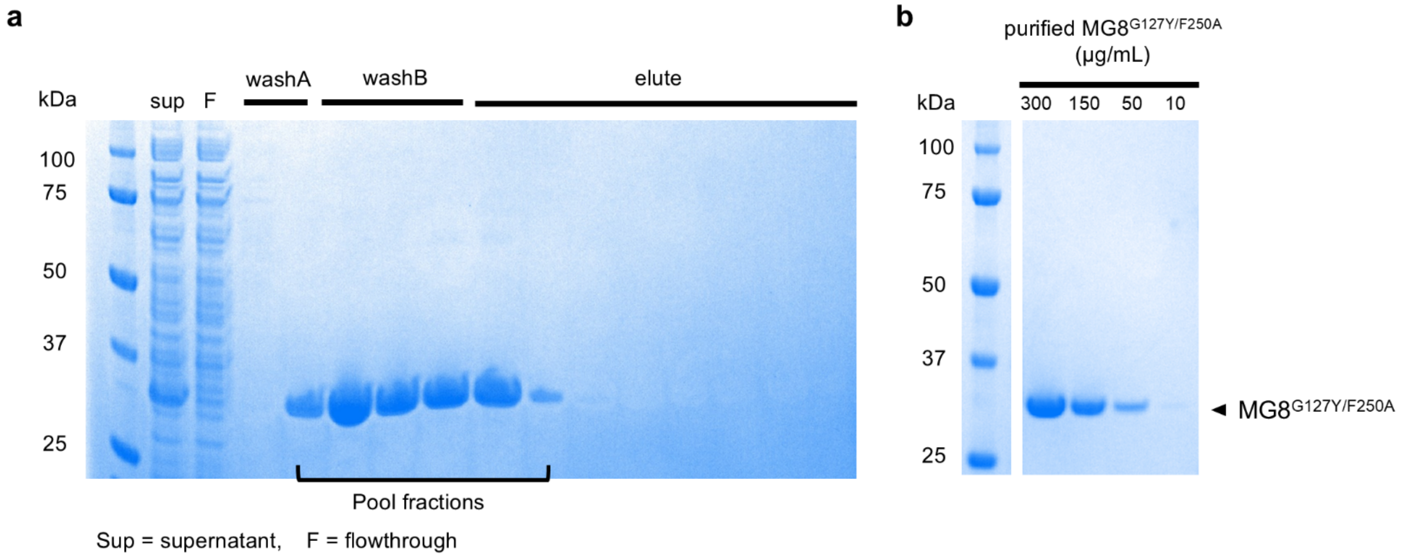

Figure S18. **a**, Secreted MG8$^{G127Y/F250A}$ could be easily purified via nickel affinity chromatography. **b**, Purity of secreted MG8$^{G127Y/F250A}$ after purification.

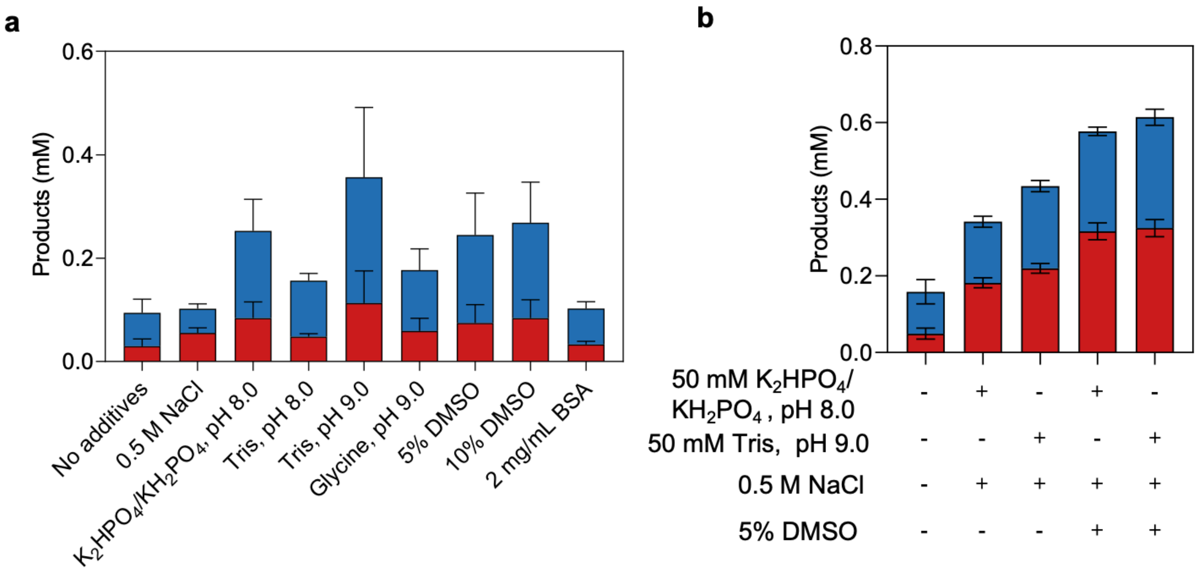

Figure S19. Optimization of cell-based PET degradation conditions. **a**, Effects of additives. BL21(DE3) was transformed with the expression plasmid for B1PelB-MG8$^{G127Y/F250A}$ and enzyme expression induced for 66 h at 25 °C. 500 µL of the culture was then supplemented with different additives (0.5 M NaCl; 50 mM K_2HPO_4/KH_2PO_4 pH 8.0; 50 mM Tris pH 8.0 and 9.0; 50 mM glycine-NaOH pH 9.0; 5-10% v/v DMSO; and 2 mg/mL BSA) and used to hydrolyze 7 mg PET powder at 37 °C for 2 days before quenching with PMSF. Generated MHET and TPA were then analyzed by HPLC. Error bars, ± S.D. from triplicates. **b**, Effects of combinations of additives. Cells and degradation of PET were prepared and analyses performed as in **a**. Error bars, ± S.D. from triplicates. Secreted MG8$^{G127Y/F250A}$ concentrations before addition of PET powder for **a** and **b** were 56 and 88 µg/mL respectively.

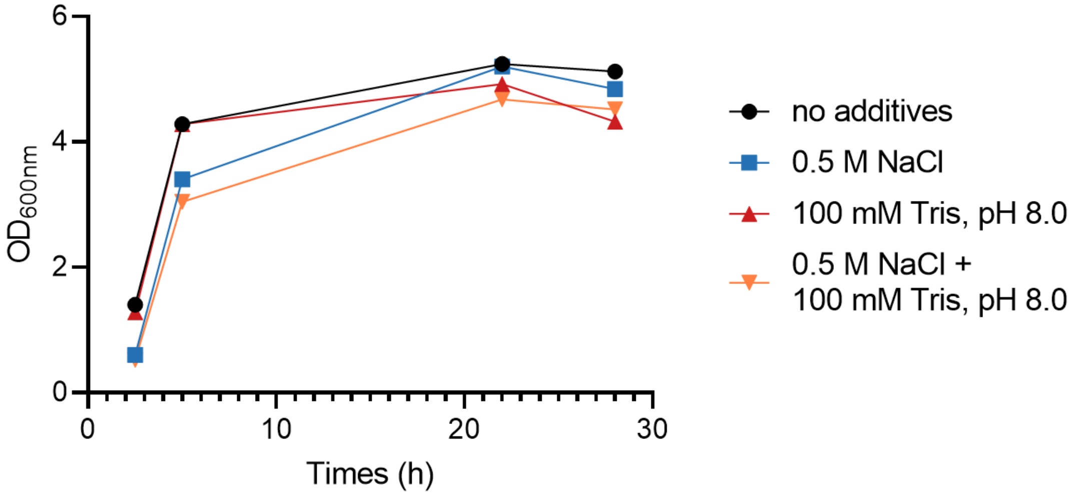

Figure S20. Growth curves of BL21(DE3) in the presence of additives to promote PET degradation. Cultures were inoculated at 5% v/v in modified M9 supplemented with 50 μg/ml kanamycin. Thereafter, cells were supplemented with different additives of 0.5 M NaCl, 100 mM Tris, pH 8.0 or both additives. Cell growth was monitored for additional 28 h.

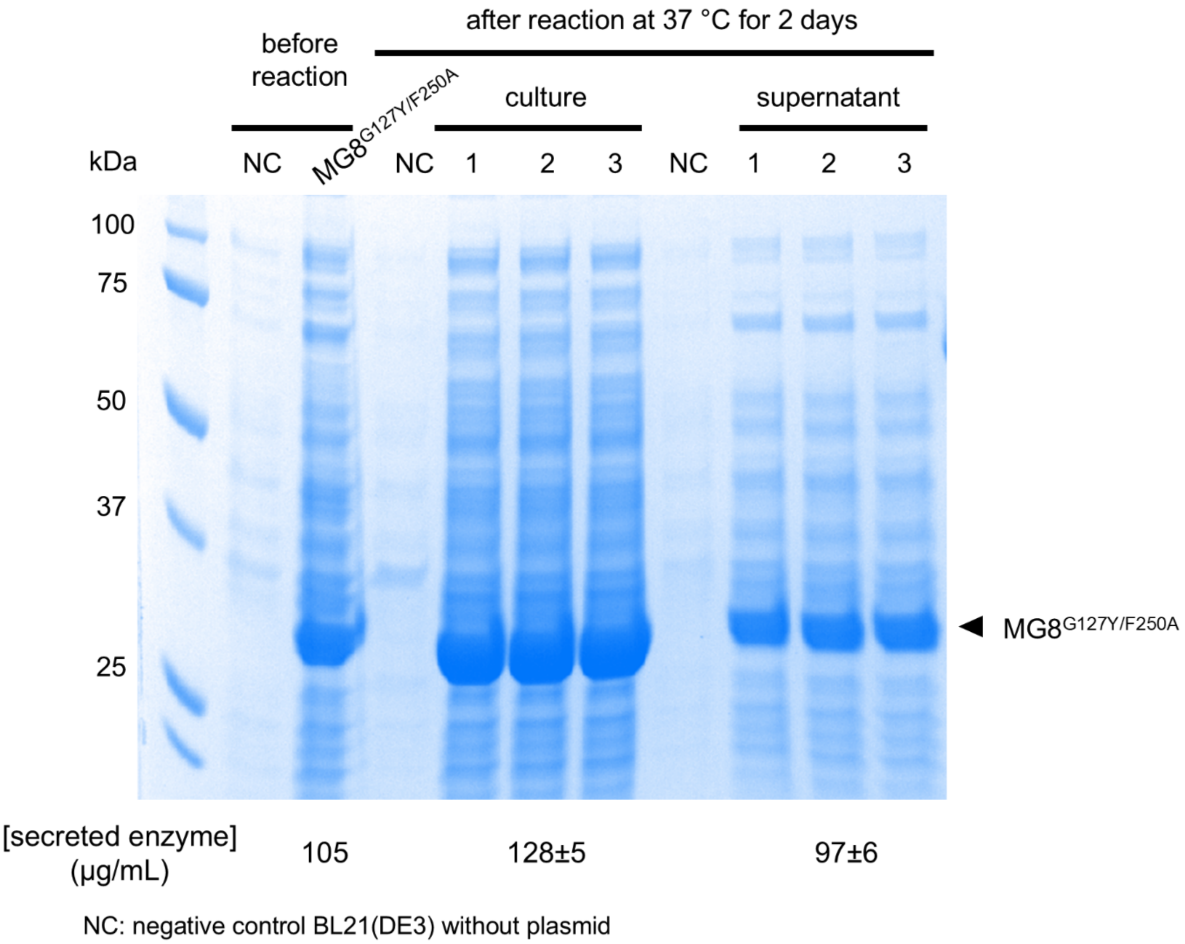

Figure S21. SDS-PAGE analysis of secreted MG8$^{G127Y/F250A}$ amount prior to the PET degradation reaction, and after 2 days of PET degradation, in the presence or absence of secretor cells. NC: negative control of *E. coli* BL21(DE3) without the MG8 expression plasmid. The "culture" contained secretor cells during PET degradation, while the "supernatant" condition only contained growth media with pre-secreted enzyme (but the secretor cells were removed by centrifugation). Triplicate results are shown for culture and supernatant conditions. MG8 size: 31.75 kDa.

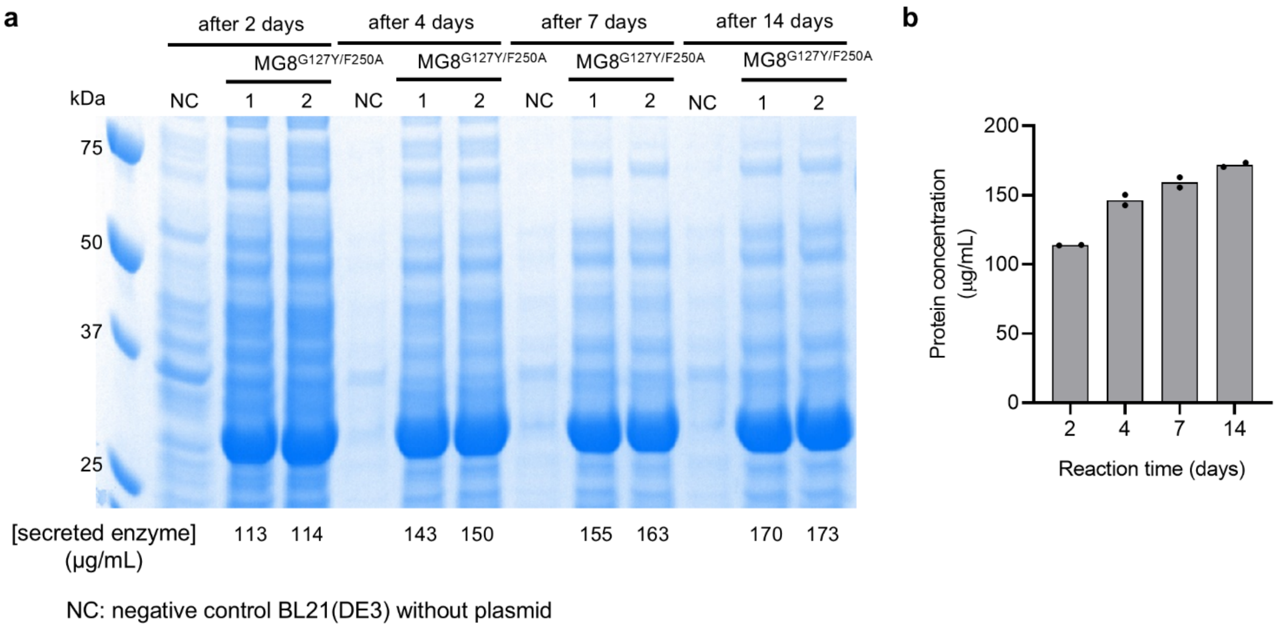

Figure S22. **a**, SDS-PAGE analysis of secreted MG8$^{G127Y/F250A}$ concentrations over time. Duplicates are shown for each time point. **b**, Secreted protein concentrations over the course of PET degradation reaction time (2-14 days).

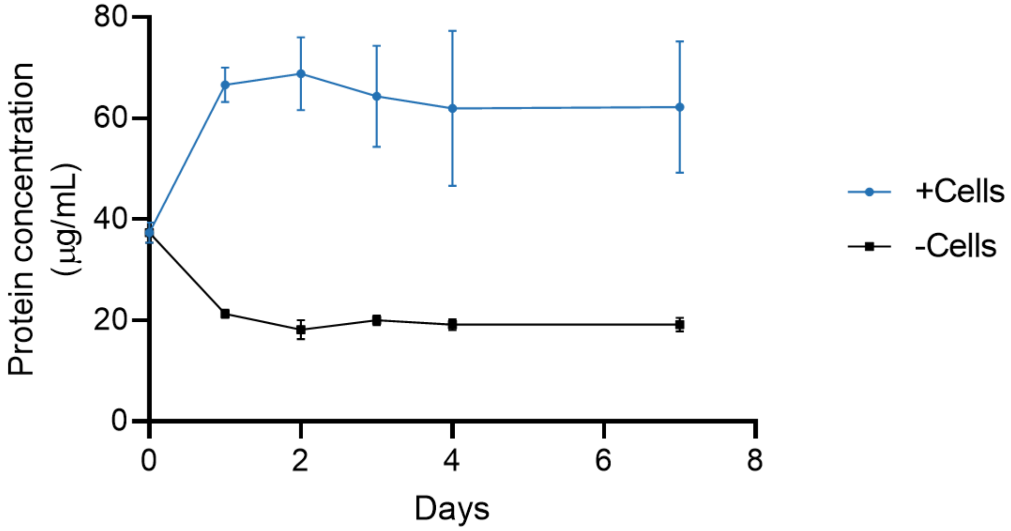

Figure S23. Concentrations of secreted MG8$^{G127Y/F250A}$ over time, with and without secretor cells. Enzymes were first expressed and secreted for 66 h at 25 °C before the PET degradation reactions at 37 °C for 1, 2, 3, 4, and 7 days. Error bars, ± S.D. from triplicates.

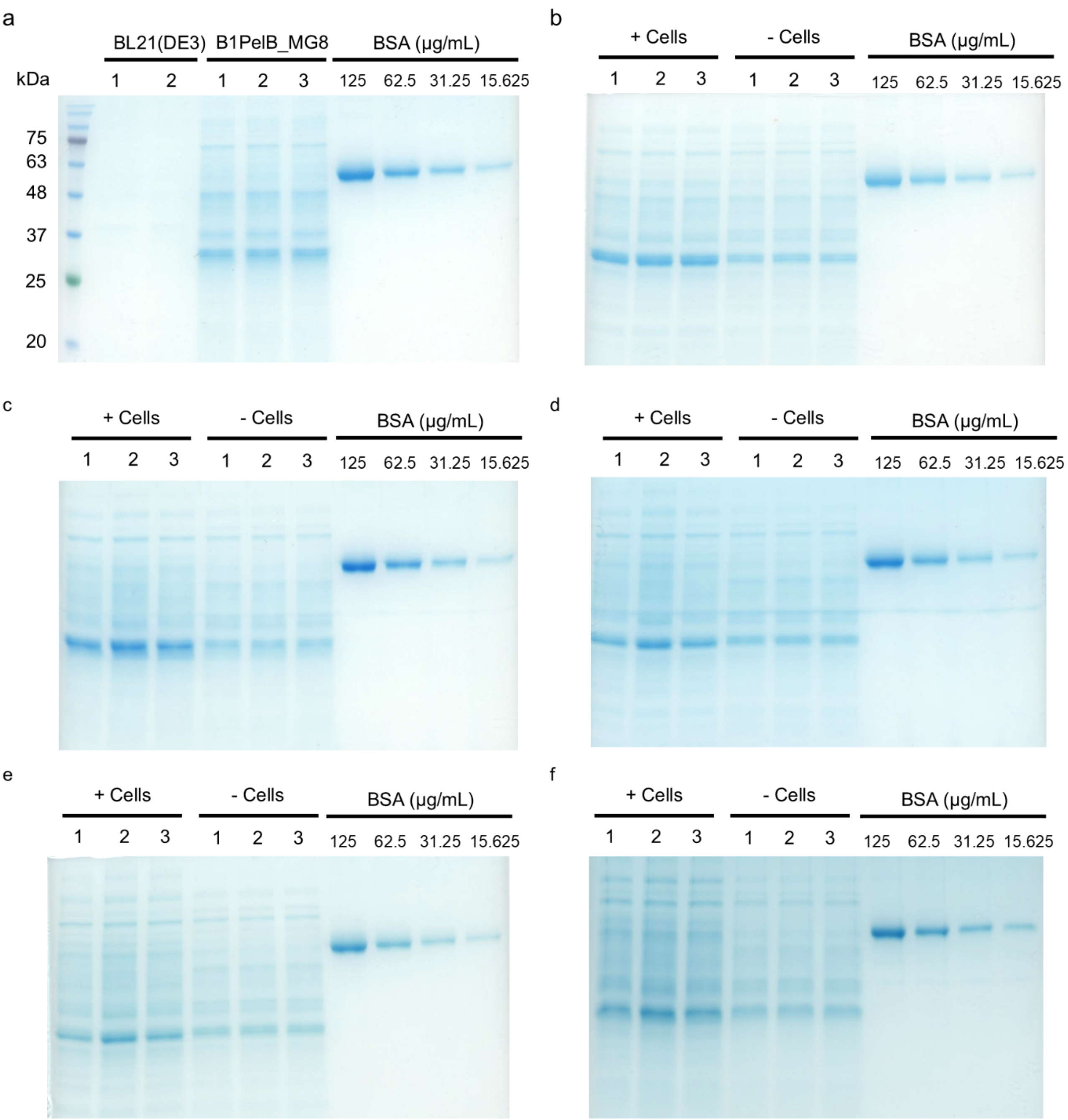

Figure S24. Representative SDS-PAGE analyses to assess concentrations of secreted MG8$^{G127Y/F250A}$ over time, with and without secretor cells. (same underlying data as **Figure S23**). **a**, MG8$^{G127Y/F250A}$ after secretion at 25 °C for 66 h. **b-f**, secreted MG8$^{G127Y/F250A}$ after PET-degrading reactions at 37 °C for 1, 2, 3, 4, and 7 days, respectively.

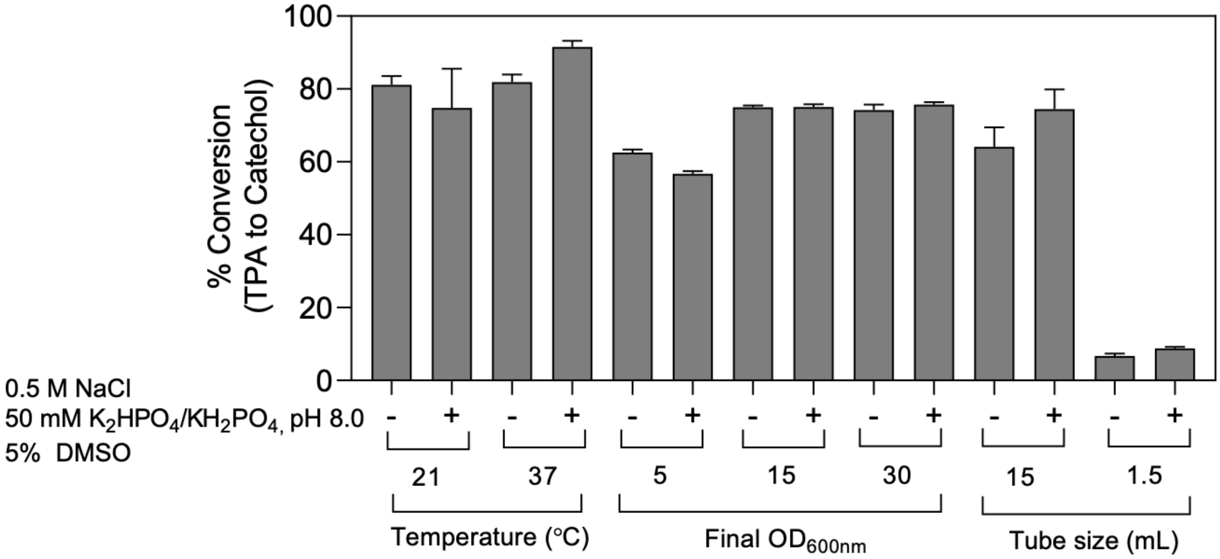

Figure S25. Optimization of catechol bioconversion conditions. Effects of culture temperature, cell density, and tube size/aeration on catechol bioconversion were assessed. The reactions were performed with 10 mM standard TPA in 1.5 mL tube or 15 mL tube, and modified M9 (0.5% w/v glucose, 1% v/v glycerol, and 0.5% w/v yeast extract) supplemented 0.5 M NaCl, 50 mM K_2HPO_4/KH_2PO_4 pH 8.0, and 5% v/v DMSO at 21 and 37 °C for 21 h. Error bars, ± S.D. from triplicates.

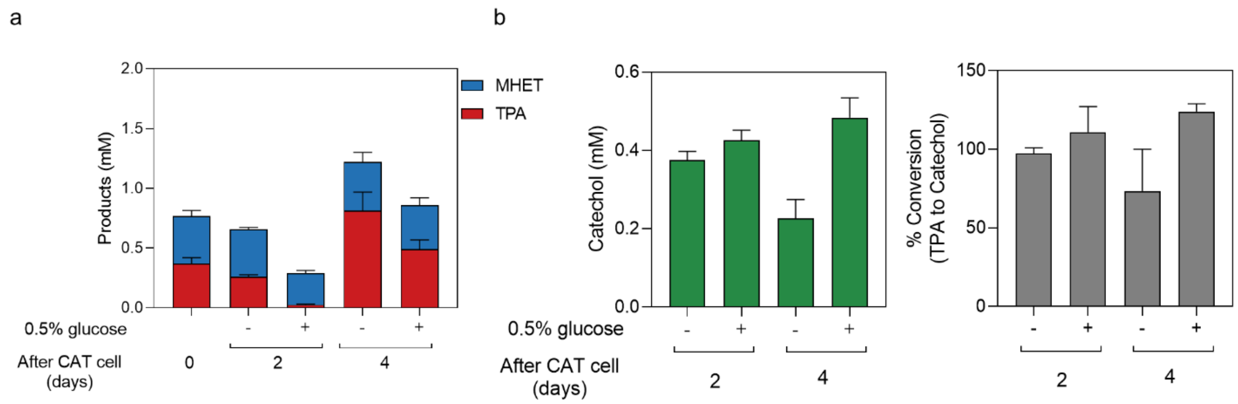

Figure S26. Effect of supplemented glucose to TPA-to-catechol conversion yields. **a**, Generated MHET and TPA from PET degradation prior to CAT-cell addition (0 day) and after 2-4 days of incubation with CAT cells, with and without 0.5% glucose supplementation. **b**, Catechol bioconversion from produced TPA after CAT-cell addition for 2-4 days. Generated catechol concentrations (mM, left) and percent conversion to catechol relative to initial TPA amount from cell-based PET degradation (right) are shown. Error bars, ± S.D. from triplicates. Secreted MG8$^{G127Y/F250A}$ concentrations before supplementation of PET powder: 69 μg/mL.

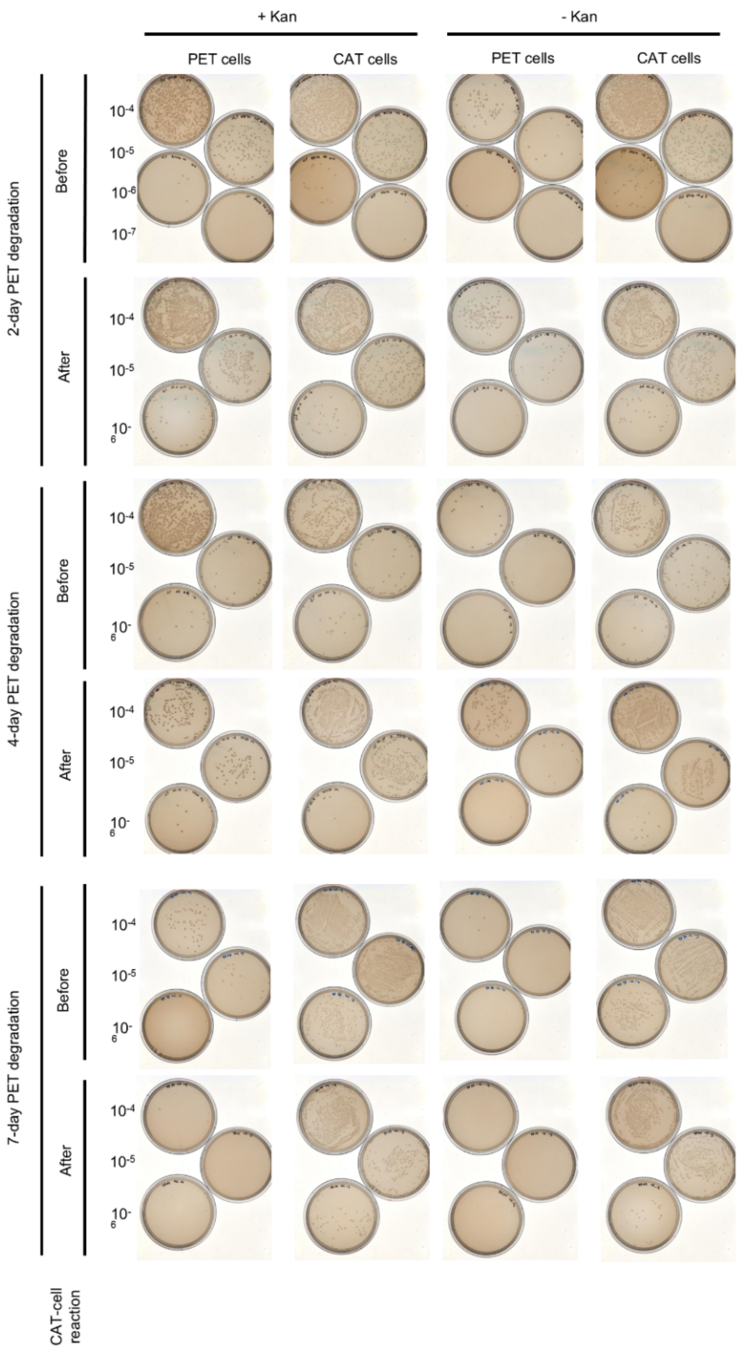

Figure S27. Assessing the presence of PET and CAT cells upon co-culturing over time. PET cells were first used to degrade PET powder at 37 °C for 2, 4, and 7 days. Thereafter, CAT cells were added to convert produced TPA to catechol at 37 °C for 2 days. The mixed cultures from before and after the 2-day reaction with CAT cells were serially diluted to final dilutions ranging from 10^{-4} to 10^{-7} and spread on LB agar supplemented with 50 μg/mL kanamycin (of which PET cells are resistant) and 100 μg/mL streptomycin (of which CAT cells are resistant). The plates were incubated at 37 °C for 16-18 h before colonies were counted. +Kan: PET-cell growth was performed in the presence of 12.5 μg/mL kanamycin. -Kan: PET-cell growth was performed in the absence of kanamycin.

Table S1. Data collection and refinement statistics of MG8 (PDB 9VZR)

Data collection	***MG8***
Synchrotron	NSRRC, Taiwan (TPS05A)
Wavelength (Å)	0.99984
Resolution range	28.83 - 1.20 (1.24 - 1.20)
Space group	P 21 21 21
Unit cell	a = 51.410, b = 61.009, c = 68.647<br>$\alpha=\beta=\gamma = 90°$
Unique reflections	66028 (6300)
Multiplicity	2.0 (2.0)
Completeness (%)	95.42 (92.26)
Mean I/sigma(I)	16.08 (2.34)
Wilson B-factor	10.05
R-meas	0.03086 (0.3688)
R-pim	0.02182 (0.2608)
CC1/2	0.998 (0.863)
Refinement statistics	
Reflections used in refinement	66028 (6291)
Reflections used for R-free	1995 (191)
R-work	0.1328
R-free	0.1604
Number of non-hydrogen atoms	2254
No. macromolecule atoms	2030
No. ligand atoms	16
No. waters	208
No. protein residues	271
RMSD (bonds) (Å)	0.0110
RMSD (angles) (°)	1.21
Ramachandran favored (%)	97.03
Ramachandran allowed (%)	2.60
Ramachandran outliers (%)	0.37
Rotamer outliers (%)	0.47
Clashscore	1.99
Average B-factors (Å^2)	13.66
Macromolecules B-factors (Å^2)	12.30
Ligands B-factors (Å^2)	33.74
Solvent B-factors (Å^2)	25.35

Table S2. Primers sequence

**Name of sequence**	Sequence
MBP-TEV-F	TACATATGAAATCTTCTCACCATCACCAT
MBP-TEV-R	TGCATTGGATTGGAAGTACAGGTTTTC
TEV-MG8-F	AACCTGTACTTCCAATCCAATGCAAATAATCCTCCTCCACCAGACGAC
TEV-MG8-R	AGGATCCTTAATTAACTAGCTGGTGCTACCCCCCCC
MG8-GFP11-R	GCGCGCCGCGATCGCGGATCCTTAATTAACTACTCGAGTGTA
71-73_Li-F	CGAACGTCTCCTCTCTCNRTNRTNRTTTTGGTGGCGGTAC
71-73_Li-R	GAGAGAGGAGACGTTCGACGTCGC
125-127_Li-F	TGTGATGACGATTGGTACCNRTNRTNRTTTTGACCAACCACCG
125-127_Li-R	GGTACCAATCGTCATCACAACAAAC
JUMP_MG8_F1_F	GATTATCGTCTCGGTCTCAAGCCAATAATCCTCCTCCACCAGACGACC
JUMP_MG8_F1_R	CGTCTCGAACGTTCGACGTCGCAACACGATA
JUMP_MG8_F2_F	CGTCTCGCGTTTCCTCTCTCGTGTCGGGCTTTG
JUMP_MG8_F2_R	CGTCTCAGGTCTCACGAACCGTACGGACAATTACCACGGTACTCCG

Table S3. Crystallinity of PET substrates

PET Type	***ΔH_m (J/g)***	***ΔH_c (J/g)***	***%Crystallinity***	***Tg***
PET film 6 mm	37.40 ± 1.74	24.60 ± 1.68	9.14 ± 0.15	76.38 ± 0.55
PET film 4 mm	36.94 ± 1.74	25.13 ± 1.26	8.43 ± 1.67	76.38 ± 0.23
PET powder 0.25 mm	38.36 ± 3.40	24.96 ± 2.11	9.56 ± 1.22	76.29 ± 0.42
R-PET film 6 mm	37.21 ± 2.42	19.78 ± 0.58	12.44 ± 1.39	76.17 ± 1.32
R-PET film 4 mm	36.45 ± 1.09	22.36 ± 1.84	10.05 ± 0.54	74.07 ± 0.65
R-PET powder 0.25 mm	37.28 ± 2.79	21.76 ± 0.52	11.08 ± 2.24	75.70 ± 0.69

Table S4. Normalized PET degradation activities of 30 best variants from the MG8(NRT$^{71-73}$/F250A) library

Plate no.	Sequence	Act/GFP 37 °C		Act/GFP 55 °C	
		MHET	TPA	MHET	TPA
**MG8^{F250A}**	**VSG**	**2679.617**	**1370.387**	**90.355**	**823.667**
P10A3	GHG	2901.240	965.787	153.242	1171.423
P10B7	GRS	708.918	369.393	25.632	355.453
P11B10	HHG	2077.473	716.183	144.513	1043.703
P11B11	YYC	n/a	n/a	n/a	n/a
P11B5	SYG	1451.520	560.591	n/a	n/a
P11C2	YRG	1463.778	916.073	70.726	634.604
P11F12	CNG	674.745	311.260	42.182	350.398
P11F9	CCG	2695.085	1152.313	168.197	1305.222
P12A5	NRR	548.791	227.024	54.623	446.440
P13C7	SYG	1331.497	687.918	69.719	491.795
P13H4	RNS	170.426	113.263	9.871	220.321
P14E5	YDG	5902.506	2489.529	165.897	1252.538
P1A3	GRS	1186.996	428.447	49.269	314.778
P1C5	RYC	1083.469	528.450	253.298	1585.983
P1D4	YGS	3035.318	914.475	235.090	1386.853
P1F5	SHG	1099.007	333.369	185.046	1162.783
P1F6	YYG	1012.719	498.683	61.887	450.431
P1G4	DCG	363.869	94.573	15.044	111.404
P3F1	SRG	856.567	345.836	43.253	257.387
P6A7	GHG	2763.980	1396.878	117.176	1074.129
P6B12	RNG	1971.192	889.185	138.191	775.080
P6C3	SND	127.064	111.633	n/a	n/a
P6D5	RDH	96.659	104.943	n/a	n/a
P7F10	NRG	2163.626	867.221	192.440	1397.764
P8A4	YDR	n/a	n/a	89.783	n/a
P8F4	CYS	722.334	509.609	112.575	997.343
P9B6	HYG	1570.956	524.256	115.747	784.677
P9C11	DNG	4301.017	1356.022	42.406	268.914
P9F7	GDR	191.481	184.394	n/a	n/a
P9H8	CSC	2769.386	1072.866	146.031	1145.731

Table S5. Normalized PET degradation activities of 30 best variants from the MG8(NRT$^{125-127}$/F250A) library

Plate no.	Sequence	Act/GFP 37 °C		Act/GFP 55 °C	
		MHET	TPA	MHET	TPA
**MG8^{F250A}**	NSG	**5072.742**	**2673.099**	**426.808**	**2893.762**
P2H11	RSG	17985.066	6588.343	1373.337	7873.817
P10F8	NSS	3181.737	921.789	124.618	710.643
P10G8	SSN	935.624	329.342	33.227	184.599
P11A7	HSR	2400.635	1158.043	72.013	521.270
P11D1	NCY	829.852	276.384	27.751	234.594
P11D11	DRY	1995.092	568.469	106.604	617.765
P11G3	YGY	2183.990	1509.415	238.671	1620.829
P12A11	CSR	3327.406	1745.530	312.055	1796.678
P12B11	NNH	3294.488	945.448	35.409	268.591
P12D8	HSS	875.995	349.690	n/a	n/a
P12E5	HSN	1649.663	671.160	159.831	986.431
P12G10	DNY	755.461	331.833	61.024	556.466
P12G7	NCY	986.107	472.201	n/a	174.314
P13C1	NSC	1981.149	712.128	163.755	1262.256
P13C8	NCY	1810.926	907.323	91.603	725.503
P13D4	NNG	2351.799	802.021	159.310	1105.683
P14B3	DSY	6064.168	1822.231	468.162	2954.610
P14F10	HNC	522.819	292.750	n/a	214.095
P14F4	DSY	6359.344	2254.891	145.062	973.248
P14G5	NSR	5541.088	2893.261	616.160	4123.166
P14G6	RNS	515.264	227.851	37.484	519.006
P14H4	NGR	1024.260	416.291	50.129	452.363
P3F7	SSY	2747.258	1054.678	117.772	684.613
P4G12	NSY	1850.070	428.096	59.408	339.713
P5C3	DSY	680.739	200.467	n/a	33.622
P8C4	DSG	662.839	486.138	n/a	n/a
P8D10	CNY	312.171	98.453	14.522	127.409
P9E6	NNR	892.982	437.572	52.383	303.520

Table S6. Comparison of percent depolymerization for PET cell-based degradation.

Enzymes	Hosts	Crystallinity (%)	Conditions	PET (mg)	Maximum TPA (mmol)	Produced TPA (mM)	Reaction volume (mL)	Produced TPA (mmol)	Depolymerization (%)	Ref.
MG8$^{G127Y/F250A}$	*E. coli* (Secretion)	9	37 °C for 14 days	7	0.0364	6.4	0.5	0.0032	8.79	**This work**
*Is*PETase	*E. coli* (Secretion)	NA	30 °C for 2 days	5	0.0260	0.85	1	0.00085	3.27	1
FAST-PETase	*E. coli* (Display)	7.58	30 °C for 4 days	5	0.0260	2.83	0.4	0.0011	4.23	2
FAST-PETase & MHETase	*S. cerevisiae* (Display)	4.2	30 °C for 7 days	9	0.0468	4.95	0.3	0.0015	3.17	3

Table S7. Comparison of percent conversion from PET for cell-based valorization.

Enzymes	Hosts	Crystallinity (%)	Conditions	PET (mg)	Maximum TPA (mmol)	Produced PCA or catechol (mM)	Reaction volume (mL)	Produced PCA or catechol (mmol)	Conversion (%)	Ref.
$MG8^{G127Y/F250A}$	*E. coli* (Secretion)	9	37 °C for 2 days	7	0.0364	1.20	0.5	0.0006	1.64	This work
$MG8^{G127Y/F250A}$	*E. coli* (Secretion)	9	37 °C for 4 days	7	0.0364	2.71	0.5	0.00135	3.72	This work
$MG8^{G127Y/F250A}$	*E. coli* (Secretion)	9	37 °C for 7 days	7	0.0364	5.66	0.5	0.0028	7.76	This work
FAST-PETase	*E. coli* (Display)	7.58	30 °C for 4 days	125	0.6509	0.7572	10	0.00757	1.16	[2]

Table S8. Number of colonies of PET and CAT cells after the sequential PET cell-based degradation of PET (2, 4, or 7 days) and CAT cell-based conversion of TPA to catechol.

PET degradation time	CAT cell supplementation	Cell strain	Dilution factor		
			10^{-4}	10^{-5}	10^{-6}
2-day degradation	Before	PET cell	>300	141	4
		CAT cell	>300	231	23
	After	PET cell	>300	284	36
		CAT cell	>300	233	26
4-day degradation	Before	PET cell	>300	31	8
		CAT cell	>300	95	23
	After	PET cell	211	74	6
		CAT cell	>300	>300	4
7-day degradation	Before	PET cell	70	14	0
		CAT cell	>300	>300	244
	After	PET cell	2	0	0
		CAT cell	>300	>300	67

Table S9. Number of colonies of PET cells—which were grown with and without supplementation of 12.5 μg/mL kanamycin during the first PET degradation step—after co-culturing with CAT cells for 2 days.

PET degradation time	CAT cell supplementation	PET cell (± Kan)	Dilution factor		
			10^{-4}	10^{-5}	10^{-6}
2-day degradation	Before	+	>300	141	4
		-	73	9	1
	After	+	>300	284	36
		-	176	21	0
4-day degradation	Before	+	>300	31	8
		-	17	3	0
	After	+	211	74	6
		-	145	7	1
7-day degradation	Before	+	70	14	0
		-	3	0	0
	After	+	2	0	0
		-	0	0	0

Materials and Methods

Molecular cloning for individual plasmids

To construct an expression plasmid for maltose-binding protein (MBP) fused to MG8 via a cleavable tobacco etch virus (TEV) protease cleavage sequence (pMBP-TEVcs-MG8), genes encoding MG8 was amplified from pET21a-MG8-GFP11 using TEV-MG8-F and TEV-MG8-R primers containing the TEVcs sequence, then subcloned into doubly digested pMBP-LbCas12a (Addgene plasmid #113431) via NdeI and BamHI sites. Transformation was performed in chemically competent *E. coli* XL1-Blue cells, and the resulting plasmid was sequence-verified via Sanger sequencing (U2Bio, Korea).

To construct pMBP-MG8^{F250A}-GFP11, the gene encoding MG8^{F250A} was amplified from pET21a-MG8^{F250A}-GFP11 using TEV-MG8-F and MG8-GFP11-R primers containing TEVcs and GFP11 sequences, then subcloned into doubly digested pMBP-LBCas12a via NdeI and BamHI sites. Transformation was performed in chemically competent *E. coli* XL1-Blue cells, and the resulting plasmid was sequence-verified via Sanger sequencing.

The JUMP modular cloning plasmid system[4] was used to construct expression plasmids for secreted MG8 variants. To domesticate MG8 into the level 0 JUMP cloning vector, MG8 genes were subcloned into a pJUMP18-Uac vector by Golden Gate assembly with BsmBI. Each segment was amplified using primers provided in Table S2. The amplicons were PCR-purified using the QIAquick PCR purification kit or gel-purified with the Monarch PCR & DNA Cleanup Kit. Subsequently, successful clones were identified via colony PCR to confirm for insertions. Thereafter, prepped plasmids were double-checked with double digestion (EcoRI and SpeI) before Sanger sequencing (Azenta). pJUMP29_ B1PelB_MG8 plasmids were assembled by BsaI with JUMP parts including T7 as the promoter, pET as the ribosome binding site, B1PelB as the N-terminus tag, His-tag as the C-terminus tag, and B00015 as the terminator. Successful clones were confirmed by colony PCR and verified for Sanger sequencing.

pPCA4 (pJUMP-*tpado-dcddh-tpaK*) will be reported in an upcoming publication by Royer, Wallace *et al* (under revision). pQlinkN-*aroY-kpdB* for the PCA to catechol conversion was previously reported[5].

Expression and purification of MBP-tagged MG8 variants

To express MBP-tagged MG8, pMBP-TEVcs-MG8 plasmid was transformed into *E. coli* BL21(DE3), then sub-cultured at 4 L volume in LB supplemented with 100 µg/ml ampicillin at 37 °C, 220 rpm. Once the OD_{600} of the culture reached 0.6-0.8, isopropyl β-D-1-thiogalactopyranoside (IPTG) was added at 1 mM (final concentration), and the culture grown for further 16 h at 18 °C. Cells was collected by centrifugation at 8,000 rpm for 20 min at 4 °C. The supernatant was discarded, and the cell pellet was resuspended in extraction buffer (25 mM Tris-HCl pH 7.5, 500 mM NaCl, and 5 mM imidazole). The cell resuspension was lysed by sonication using 40% pulse amplitude (on 5 s and off 10 s) until completely lysed. The lysate was centrifuged at 15,000 rpm for 30 min at 4 °C. The resulting supernatant was applied to Chelating Sepharose^{TM} Fast Flow column which was

pre-loaded with 200 mM $NiSO_4$ and pre-equilibrated with binding buffer (25 mM Tris-HCl pH 7.5, 500 mM NaCl, 5 mM imidazole). The column was washed with 5 CV of binding buffer, 10 CV of washing buffer (25 mM Tris-HCl pH 7.5, 500 mM NaCl, 40 mM imidazole) and eluted with 7 CV of elution buffer (25 mM Tris-HCl pH7.5, 150 mM NaCl, 500 mM imidazole). The eluted fractions containing His_{10}MBP-MG8 were pooled, and a fusion tag removed with TEV protease using 20:1 mg of protein:TEV protease enzyme ratio in TEV cleavage buffer (50 mM Tris-HCl pH 7.5, 0.5 mM EDTA, 1 mM DTT, 5% Glycerol). The reaction mixture was gently agitated at 20 °C overnight, diluted with 25 mM sodium phosphate pH 6.0 to conductivity of 3-5 mS/cm, then adjusted to pH 6.0 with 1 M phosphoric acid. Subsequently, the protein solution was applied to a SP Sepharose FF column (XK26 x 100 mm), which was pre-equilibrated with binding buffer (25 mM sodium phosphate pH 6.0). The column was washed with 10 CV of binding buffer and eluted with 10 CV of elution buffer (50 mM sodium phosphate pH 6.5, 50 mM sodium chloride). The purified MG8 fractions were pooled, concentrated, and exchanged into storage buffer (25 mM Tris-HCl pH 7.5, 150 mM NaCl) using 10 kDa Amicon centrifugal filter (Merck Millipore) and stored at -80 °C.

Protein crystallization, data collection and structural determination

The crystallization experiments were set up at 19 °C using the sitting-drop vapor-diffusion method in Swissci 96-Well 2-Drop MRC crystallization plates (Molecular Dimensions, Sheffield, UK) using a Mosquito crystallization robot (SPT Labtech, Melbourn, UK). For crystallization screening of MG8, freshly prepared protein solution (16 mg·mL^{-1} in 25 mM Tris-HCl pH 7.5, 100 mM NaCl) was mixed with the screening solutions of the Structure Screen I-II (#MD1-01 and #MD1-02), PACT premier (#MD1-29) and Morpheus kits (#MD1-46) (Molecular Dimensions, UK) with protein solution: mother liquor ratios of 150 nl:200 nl in the top wells and 200 nL:200 nL in the bottom wells. We obtained crystals of MG8 in the drops containing 200 mM sodium chloride, 100 mM sodium acetate pH 5.0, 20%(v/v) PEG6000 after incubation for 23 weeks.

The X-ray diffraction experiment of MG8 were performed at the TPS-05A beamline of the National Synchrotron Radiation Research Center (NSRRC) in Hsinchu, Taiwan. The diffraction images were processed using HKL2000[6]. The structure of PE-H[7] (PDB: 6SBN) was used as a template for molecular replacement (MR). The MR phasing technique was conducted by PHASER in Phenix program suit[8]. The structure was then refined and rebuilt in *Phenix refine*[8] and *coot*[9], respectively (Table S1). The final structure was displayed using PyMol[10]. The atomic coordinated data was deposited in the Protein Data Bank (PDB accession code 9VZR).

Molecular dynamics simulations

All MD simulations were conducted using GROMACS (version: 2024.2[11]) with CHARMM36[12] force field. The crystal structures of MG8 and FAST-PETase (PDB: 9VZR and 7SH6 respectively) were embedded within a cubic box of solutes with a side length of 12 Å. Water molecules (model: TIP3P) were added as solutes. Then, salt ions of NaCl

were added at the designated NaCl concentration. The overall charge of system was neutralized by adding additional Na^+ or Cl^- counter ions to the solvent box.

A short energy minimization step was performed for each system with a maximum of 50,000 steps and a maximal force of 1000 kJ $mol^{-1}nm^{-1}$. After the energy minimization step, the systems were equilibrated in a position-restrained NVT simulation. The temperature was gradually increased to the desired temperature (310 K or 328 K) over 100 ps. Subsequently, the system underwent equilibration under NPT for 100 ps. MD production simulations were performed via the LINCS algorithm[13] for 100 ns. The time step was set at 2 fs. The simulation of each system was performed in triplicate, in which each replicate was subjected to its own equilibration energy from the NVT simulation. The RMSD and RMSF of each system were then calculated using GROMACS.

DNA Library construction

Site-saturated libraries of $MG8^{F250A}$ containing mutations at the β1–β2 loop (residue 71-73) and the β4– α3 loop (residue 125-127) were constructed using overlap-extension PCR with degenerate primer pairs bearing the NRT codon at the desired positions (71-73_Li primers for the β1–β2 loop and 125-127_Li primers for the β4– α3 loop; primer sequences in Table S2) and pMBP-$MG8^{F250A}$-GFP11 as the template. Transformation was performed in electrocompetent *E.coli* XL1-Blue cells. Sequence diversity within the two loop libraries was assessed by Sanger sequencing of 20 random clones for each library, as well as by pooled sequencing.

Enzyme production for library screening

pMBP-MG8-GFP11 libraries were expressed in competent *E.coli*_BL21(DE3) cells. Single colonies were used to inoculate 400 µL LB media supplemented with 100 µg/mL ampicillin in 96-well 2 mL deep-well plates. Each plate contained one positive control of BL21(DE3) transformed the template plasmid (pMBP-$MG8^{F250A}$-GFP11); two negative control replicates of BL21(DE3) transformed with pMBP-LbCas12a; and two negative control replicates of LB media containing 100 µg/mL ampicillin. Plates were incubated overnight at 37 °C in a shaking incubator (220 rpm). Expression cultures were then prepared by inoculating 400 µL of LB media containing 100 µg/mL ampicillin with 20 µL of the overnight culture in fresh 96-well deep-well plates. The inoculated plates were incubated at 37°C, 220 rpm for 2-3 hours before induction by the addition of 1 M IPTG stock solution (at ~1:1000 IPTG: culture volume ratio, to give 1 mM final IPTG concentration). Plates were further incubated for 24 hours at 16 °C and 220 rpm. Cells were harvested by centrifugation at 3,900 rpm for 20 minutes. Resulting pellets were resuspended in 36 µL 1X BugBuster® 10X Protein Extraction Reagent (Merck Millipore, #70921) containing 10 µg/mL DNase I. Cells was lysed for 40 minutes at 37°C and 220 rpm before 84 µL of the reaction buffer (25 mM Glycine, 10 mM NaCl; pH 9.0) was added. Insoluble cell debris was removed by centrifugation for 30 minutes at 3,900 rpm.

Library activity assay on PET film

The cell lysates were transferred to 96-well plates (Corning,#3595) and centrifuged for 40 minutes to remove insoluble fractions. Then, 20 µL of clarified lysates were transferred to 96-well plates with 150 µL reaction buffer (25 mM Glycine, 10 mM NaCl; pH 9.0) and a PET disc (9% crystallinity as assessed by differential scanning calorimetry) with a 6 mm diameter in each well. Plates were sealed with clear tape and incubated with incubator shaker at 220 rpm under two different temperatures, 37°C and 55°C, for 48 hours. The reactions were quenched by addition of 15 µL of 160 mM phosphate buffer pH 2.5 containing 0.1% formic acid, then incubated at 37 °C for 30 min. Protein precipitates were removed by centrifugation at 3900 rpm for 30 min. The clarified reaction was transferred to 96-well microtiter plates (Corning,#3595) for injection into the RapidFire 365 mass spectrometry system.

High-throughput activity screening with RapidFIRE

High-throughput mass spectrometric analyses were carried out on the RapidFire 365 mass spectrometry system (Agilent Technologies). 10 µL of clarified reactions were loaded onto a C18 cartridge (4 µL, type C, #G9205A, Agilent Technologies). Water (A) and 100% acetonitrile (B) were used as mobile phase solutions. 100% of mobile phase A was used in the loading and extra washing steps (15% B) were performed for 800 ms at the flow rate of 1.5 and 1.25 mL/min respectively. The elution was done with 25% B for 25,000 ms at 0.4 mL/min flow rate. The total time for an injection per sample was 80 sec. The multiple reaction monitoring (MRM) mode was used for the mass spectrometry detection in the negative ionization mode. The parental ion of MS1 ([M + H]-) and the product ion of MHET were 209 and 165 respectively. MS1 ([M + H]-) and the product ion for TPA were 165 and 121 respectively. The fragmentor voltage and collision energy of two ions were 72 V and 11 eV, respectively. To specify the MHET/TPA molecule, the signal for the more abundant transitions were obtained at the second optimized collision energy. The precursor ions of MHET could produce the product ions of m/z at 121 as same as the parameters above. Whereas the signal transition m/z 121 >> 77 for TPA were extracted with the fragmentor voltage and collision energy of 72 V and 27 eV, respectively.

Split GFP reconstitution for enzyme expression level measurements

20 µL of cell lysates were mixed with a solution of the purified GFP1-10 domain in a buffer containing 100 mM Tris–HCl, 150 mM NaCl and 10% glycerol pH 7.4, and incubated overnight at 37 °C. The resulting reconstituted GFP fluorescence was measured on a microplate reader (Infinite®200 PRO, Tecan) (λ_{ex} = 486 nm/λ_{em} = 515 nm).

PET depolymerization assay with purified enzymes

Selected MG8 variants were expressed in BL21 (DE3) and purified as previously reported[14]. PET degradation activities of purified enzyme variants were tested against various PET substrates: 6-8 mg PET cup lid (9% crystallinity), 6-8 mg recycled PET food packaging (10% crystallinity), and 10 mg PET powder (30% crystallinity). Enzymatic PET

degradation was performed in 96-well plates with 0.5 μM enzyme and the desired substrate in a reaction buffer (25 mM Glycine pH 9.0 and 0.01 – 4 M of NaCl) at 37 or 55 °C for 48 hours. The reactions were quenched by 160 mM phosphate buffer pH 2.5 and 0.1% formic acid and incubated for 30 min at 37 °C. Protein precipitate was removed by centrifugation at 3900 rpm for 30 min and the clarified reactions was transferred to UV-transparent microplates (Corning, #3635) for absorbance monitoring at 260 nm using Infinite®200 PRO microplate reader (Tecan). Specific PET degradation products were further quantified using HPLC (Shimadzu, LC-2050) equipped with a reverse-phase Agilent Eclipse Plus C18 column (4.6 x 250 mm, 5 μm), heated in a column oven to 30 °C. Ultrapure water with 0.1% formic acid (A) and acetonitrile with 0.1% formic acid (B) were used as mobile phase with a fixed flow rate 1.5 $mL \cdot min^{-1}$. 10 μL of samples was injected into the column followed by 20% of mobile phase B. To separate TPA and MHET, an 8-min gradient of 20-40% mobile phase B was used, followed by a 2-min gradient of 40 - 100% mobile phase B to clean the column. The column was then equilibrated back to 20% mobile phase B. Retention times of TPA and MHET were characterized using standards of commercial TPA (TCI) and enzymatically generated MHET[14]. Calibration curves for TPA and MHET at 3, 2, 1, 0.1 and 0.01 mM were also generated as previously reported[14].

To compare $MG8^{G127Y/F250A}$ activity with different PET particle sizes, enzyme activity assays were set as described above using PET cup lid (ϕ = 6 mm) and ~8 mg of cryo-milled PET (with sieve size 4 mm and 0.25 mm). 10% v/v DMSO was also optionally added to the reaction buffer. The enzyme activity was quantified by monitoring total PET degradation products via absorbance at 260 nm using Infinite®200 PRO microplate reader (Tecan).

Melting temperature characterizations

The melting temperatures (T_m) of MG8 variants were determined using differential scanning fluorimetry (DSF). 20 μL of 1.5 μM protein solutions were prepared in the reaction buffer (25 mM Glycine pH 9.0 and 0.01, 0.15, 0.5, 1, or 4 M NaCl) with 2 μL of 10X SYPRO Orange dye stock solution (Thermo Fisher) in optically clear PCR tubes (Biorad). DSF melting analyses were carried out on a Biorad CFX Connect Real-Time PCR detection system. The temperature was set to increase from 25 °C to 95 °C, and the melting temperature was determined from the peak of the first derivatives. Each T_m value corresponded to a mean T_m from three replicates.

Crystallinity characterization of PET substrates (Table S3)

The percentage of crystallinity of PET substrates was characterized by a differential scanning calorimeter (PerkinElmer, DSC 8500 double furnace). PET film or powder (5-7 mg) was placed in a standard aluminum pan with cover. The temperature of the sample was held at 0 °C for 1 min, then increased from 0 °C to 270 °C at the rate of 10 $°C \cdot min^{-1}$, and held at 270 °C for 1 min. Subsequently, the temperature was reduced from 270 °C back to 0 °C, at rate of 100 $°C \cdot min^{-1}$. The cycle of heating and cooling was repeated once

more. The percentage of crystallinity was calculated using the data obtained from the first heating using the following equation:

$$\%Crystallinity = (\Delta H_m - \Delta H_c)/\left(\Delta H_{(m^{\circ})}\right)$$

Where ΔH_m is the enthalpy of melting (J·g^{-1}), ΔH_c is the enthalpy of cold crystallization (J·g^{-1}), and $\Delta H_{m^{\circ}}$ is the reference value of enthalpy of melting for 100% crystalline PET (140.1 J·g^{-1}). The enthalpy values were obtained by integrating peak areas.

Expression and secretion of B1PelB-MG8 variants

Our optimal expression and secretion conditions for MG8 are as follows: to express B1PelB-MG8-6xHis, the pJUMP29 plasmid containing the gene was transformed into *E. coli* BL21(DE3). A single colony was picked and cultured in 5 mL LB medium with 50 µg/mL kanamycin at 37 °C, 220 rpm for 16–18 hours. The culture was then inoculated at 5% (v/v) into modified M9 medium (supplemented with 0.5% w/v glucose, 1% w/v glycerol, and 0.5% w/v yeast extract) with 50 µg/mL kanamycin. Cells were grown at 37 °C, 220 rpm for 3 hours until reaching an OD_{600nm} of 0.6–0.8. Protein expression was induced by adding IPTG to a final concentration of 0.25 mM, followed by incubation at 25 °C, 220 rpm for 66 hours.

For Figure 4a (assessment of secretion efficiency of different MG8 variants), we induced cells with 1 mM IPTG and incubated at 18 °C for 48 h.

For Figure S17a, the induction condition was performed at a fixed temperature of 18 °C and varying expression times (18, 24, 42, 48, and 66 h).

For Figure S17b, the induction condition was varied (IPTG concentration of 0.1, 0.25, 0.5, or 1 mM) at 25 °C for 66 h.

For Figure S17c, the secreted conditions were conducted at varying temperatures (18, 25, and 30 °C) at a fixed incubation time of 48 h.

For Figure S17d, the secreted conditions were performed in different media: modified M9 supplemented 0.5% w/v glucose, 1% v/v glycerol, and 0.5% w/v yeast extract; Luria broth, LB; or terrific broth, TB) at a fixed temperature of 25 °C for 66 h with 1 mM IPTG.

For SDS-PAGE sample preparation, 20 µL of the supernatant was mixed with 7 µL of SDS loading dye, heated at 85 °C for 5 min, and loaded into SDS-PAGE for 20 µL per well. A standard curve using BSA standards (typically at concentrations of 125, 62.5, 31.25, and 15.625 µg/mL) was generated to convert observed band intensities of MG8 into concentrations via analysis with a Bio-Rad gel imager.

Purification of secreted MG8 variants

The secreted MG8 was expressed at optimal conditions as previously described. The culture supernatant was separated by centrifugation at 10,000 rpm for 30 minutes and then loaded onto a 4 mL HiPur™ Ni-NTA resin column pre-equilibrated with the binding buffer (25 mM Tris-HCl, 500 mM NaCl, pH 7.5). The column was washed with the binding

buffer for 3 column volumes (CV), followed by an additional wash with the binding buffer containing 40 mM imidazole for 7 CV. MG8 was then eluted using the binding buffer supplemented with 400 mM imidazole for 7 CV. Protein-containing fractions were pooled based on SDS-PAGE analysis, and imidazole was removed using a desalting column into storage buffer (25 mM Tris-HCl, pH 7.5, 500 mM NaCl). Purified proteins were stored at -20 °C and -80 °C.

Cell growth assays

The cell growth was monitored by OD_{600nm} for 2, 4, 22, and 24 h. The culture was inoculated at 5% v/v into modified M9 medium supplemented with 50 µg/ml kanamycin and different additives (0.5 M NaCl; 0.1 M Tris, pH 8.0; or both additives), followed by incubated at 37 °C

Cell-based PET degradation

For PET degradation, 500 µL of culture, expressed under optimal conditions at 25 °C for 66 h, was transferred into a 1.5 mL tube and supplemented with 7±1 mg of PET powder (0.25 mm sieve, sourced from a coffee cap) along with 0.5 M NaCl, 50 mM K_2HPO_4/KH_2PO_4 (pH 8.0), and 5% DMSO. The reaction mixture was then incubated at 37 °C for 2 days.

The reactions were centrifuged at 18,000 xg for 10 min, and supernatants were quenched with PMSF (final concentration 5 mM) for 30 min, then centrifuged under the same condition. Each supernatant was transferred to a new 1.5 mL tube and diluted 6-time with 50 mM Tris-HCl, pH 7.5, 150 mM NaCl, and analyzed with HPLC. The diluted reaction was analyzed on a Vanquish HPLC equipped with a reverse-phase Hypersil GOLD, 3 mm diameter x 150 mm length, C18 column, heated in a column oven to 30 °C. The method was performed with a 6-min gradient of 20-40% acetonitrile with 0.1% TFA in water at 0.4 mL/min flow rate, followed by a wash with 90% acetonitrile added 0.1% TFA in water for 3 min and column equilibration with 20% acetonitrile for 5 min. In these reactions, we specifically monitored the hydrolysis products mono(2-hydroxyethyl) terephthalate (MHET) and terephthalic acid (TPA). TPA calibration curve was generated using commercial TPA at concentrations of 0.75, 0.5, 0.25, and 0.1 mM. MHET calibration curve was generated using *Is*PETase-mediated hydrolysis of BHET. *Is*PETase hydrolyzed BHET to 99% MHET and 1% TPA, as previously reported[14]. MHET concentrations used for calibration curves were 0.75, 0.5, 0.25, and 0.1 mM.

For the comparison of presence and absence of the secretor cells (Figure 4b), 500 µL of secreted MG8 after the optimal secretion condition was transferred into a 1.5 mL tubes and supplemented with ~7 mg of PET powder, 0.5 M NaCl, 50 mM K_2HPO_4/KH_2PO_4, pH 8.0, and 5% v/v DMSO, followed by incubation at 37 °C and 220 rpm for 2 days. For purified MG8, 7 mg PET powder was incubated with 3 µM purified MG8 with 0.5 M NaCl, 50 mM K_2HPO_4/KH_2PO_4, pH 8.0, and 5% v/v DMSO in a 1.5 mL tubes at 37 °C and 220 rpm for 2 days.

For Figure 4c, 500 µL of secreted MG8 culture was transferred into 1.5 mL tubes and supplemented with 7±1 mg of PET powder, 0.5 M NaCl, 50 mM K_2HPO_4/KH_2PO_4, pH 8.0, and 5% v/v DMSO, followed by incubation at 37 °C and 220 rpm for 2, 4, 7, and 14 days. For purified MG8, 7 mg PET powder was incubated with 3 µM purified MG8 in 0.5 M NaCl, 50 mM K_2HPO_4/KH_2PO_4, pH 8.0, and 5% v/v DMSO in 1.5 mL tubes at 37 °C and 220 rpm for 2, 4, 7, and 14 days.

To optimize additives (Figure S19a), 500 µL of secreted MG8 culture was transferred into a 1.5 mL tube and supplemented with ~7 mg of PET powder and different additives including 0.5 M NaCl, DMSO (5 and 10% v/v), BSA (2 mg/mL), and different buffers (50 mM K_2HPO_4/KH_2PO_4, pH 8.0; 50 mM Tris, pH 8.0 and 9.0; and 50 mM glycine, pH 9.0). The reactions were then incubated at 37 °C and 220 rpm for 2 days. The additives stock was prepared as follows: 4 M NaCl, 100% DMSO, 1 M buffers, 100 mg/mL BSA.

For Figure S19b, 500 µL of secreted MG8 culture was transferred into a 1.5 mL tube and supplemented with ~7 mg of PET powder and a combination of additives from 0.5 M NaCl, buffer (50 mM K_2HPO_4/KH_2PO_4, pH 8.0, and 50 mM Tris, pH 9.0), and 5% v/v DMSO. The reactions were then incubated at 37 °C and 220 rpm for 2 days.

Bioconversion of TPA to catechol

To prepare CAT cells, pPCA4_tphA1, tphA2, tphB2, DCDDH, tpaK, and pAroY/KpdB were transformed into *E. coli* BL21(DE3) and cultured in 5 mL LB medium containing 100 µg/mL carbenicillin and 50 µg/mL streptomycin at 37 °C, 220 rpm for 16–18 h. The culture was then inoculated at 2% v/v into fresh LB medium supplemented with the same antibiotics and incubated at 37 °C, 220 rpm for 2 h and 20 min, until OD_{600} reached 0.6–0.8. Expression was induced with 1 mM IPTG, followed by further incubation at 30 °C, 220 rpm for 16–18 h. The cells were harvested and stored at -20 °C until use.

For optimization of conversion to catechol, 150 mM commercial TPA stock solution was prepared in dimethyl sulfoxide. To set up 500 µL reaction, 10 mM TPA was dissolved in modified M9 medium without an antibiotic and added to CAT cells at different OD_{600} of 5, 15, or 30, with and without supplemented 0.5 M NaCl, 50 mM K_2HPO_4/KH_2PO_4, pH 8.0, and 5% v/v DMSO. Subsequently, the reaction was transferred to a 1.5 or 15 mL tube and incubated horizontally at 21 and 37 °C for 21 h. The reaction mixture was diluted 3-fold in 100 mM HCl, vortexed for 10 s, and centrifuged at 18,000 × g for 10 min. The supernatant was analyzed via HPLC. The HPLC conditions included an initial 5-min isocratic run with 5% acetonitrile + 0.1% v/v TFA, followed by a 5-min gradient from 5% to 15% acetonitrile, a 10-min gradient from 15% to 50% acetonitrile, and column re-equilibration with 5% acetonitrile for 5 min.

To combine PET cells and CAT cells (Figure S26), 500 µL of secreted MG8 culture (after optimal expression and with 12.5 µg/mL kanamycin) was transferred into 1.5 mL tubes and supplemented with ~7 mg of PET powder, 0.5 M NaCl, 50 mM K_2HPO_4/KH_2PO_4, pH 8.0, and 5% v/v DMSO. The PET degradation reaction was performed at 37 °C and 220 rpm for 2 days. Supernatants were collected from the resulting reactions, and degradation

products quantified by HPLC. 500 µL of the reactions were further mixed with CAT cells at OD_{600} of 15 supplemented with 0.5% w/v glucose. The cell mixtures were grown via horizontal shaking at 37 °C for 2 and 4 days. After incubation, each reaction mixture was diluted 3-fold in 100 mM HCl, vortexed for 10 s, and centrifuged at 18,000 × g for 10 min. The supernatant was analyzed via HPLC, following the previously described method. The chromatographic conditions included an initial 5-min isocratic elution with 5% acetonitrile + 0.1% TFA, followed by a 5-min gradient from 5% to 15% acetonitrile, a 10-min gradient from 15% to 50% acetonitrile, and column equilibration with 5% acetonitrile for 5 min.

For Figure 5b-c, the PET degradation was performed at 37 C° for 2, 4, and 7 days before the CAT-cell addition at 37 °C for 2 days.

For Figure S27, PET cells were cultured with and without supplemented 12.5 µg/mL kanamycin during the initiation expression and secretion step. Subsequently, PET cells were used for PET degradation as previously described as Figure 5b-c.

Calculation of the degree of depolymerization and the conversion percentage

For Table S6, the degree of depolymerization of PET plastic was calculated as the ratio of produced TPA (mmol) to theorical maximum of TPA from PET with the following equation:

$$\%Depolymerization = \frac{produced\ TPA\ (mmol)}{theoreical\ maximum\ of\ TPA\ (mmol)} \times 100$$

$$Theoreical\ maxmimum\ of\ TPA\ (mmol) = \frac{PET\ substrate\ (mg) \times 0.8652}{166.13\ \frac{g}{mol}}$$

where 0.8652 is the theoretical mass yield coefficient of TPA from PET[15], calculated as the ratios of MW of TPA (166.13 g/mol) to the MW of repeating units of PET (192 g/mol).

For Table S7, the degree of conversion of PCA or catechol from PET was calculated similarly to the degree of depolymerization, with the produced PCA or catechol replacing TPA.

Serial dilution-based isolation

For Figure S27 and Tables S8-9, PET cells were cultured with or without 12.5 µg/mL kanamycin during the initial growth step. Subsequently, the PET degradation reaction was performed at 37 °C for 2, 4, and 7 days. The resulting reactions were resuspended with CAT cells as previously described at 37 °C for 2 days. The mixed culture of PET cells

and CAT cells from PET degradation reaction on day 2, 4, and 7 were serially diluted to final dilutions ranging from 10^{-4} to 10^{-7} and spread on LB agar supplemented with 50 µg/mL kanamycin (of which PET cells are resistant) and 100 µg/mL streptomycin (of which CAT cells are resistant). The plates were incubated at 37 °C for 16-18 h before colonies were counted.

References

1 Shi, L., Liu, H., Gao, S., Weng, Y. & Zhu, L. Enhanced Extracellular Production of IsPETase in Escherichia coli via Engineering of the pelB Signal Peptide. *Journal of Agricultural and Food Chemistry* **69**, 2245-2252 (2021). https://doi.org:10.1021/acs.jafc.0c07469

2 Zheng, S., Xu, X., Gao, T. & Song, H. One-Pot Microbial Cell Factory Strategy for the Production of Protocatechuic Acid from Polyethylene Terephthalate Waste. *ACS Sustainable Chemistry & Engineering* **12**, 5632-5639 (2024). https://doi.org:10.1021/acssuschemeng.4c00363

3 Gulati, S. & Sun, Q. Complete Enzymatic Depolymerization of Polyethylene Terephthalate (PET) Plastic Using a Saccharomyces cerevisiae-Based Whole-Cell Biocatalyst. *Environmental Science & Technology Letters* **12**, 419-424 (2025). https://doi.org:10.1021/acs.estlett.5c00190

4 Valenzuela-Ortega, M. & French, C. Joint universal modular plasmids (JUMP): a flexible vector platform for synthetic biology. *Synth Biol (Oxf)* **6**, ysab003 (2021). https://doi.org:10.1093/synbio/ysab003

5 Valenzuela-Ortega, M., Suitor, J. T., White, M. F. M., Hinchcliffe, T. & Wallace, S. Microbial Upcycling of Waste PET to Adipic Acid. *ACS Central Science* **9**, 2057-2063 (2023). https://doi.org:10.1021/acscentsci.3c00414

6 Otwinowski, Z. & Minor, W. Processing of X-ray diffraction data collected in oscillation mode. *Methods Enzymol* **276**, 307-326 (1997). https://doi.org:10.1016/s0076-6879(97)76066-x

7 Bollinger, A. *et al.* A Novel Polyester Hydrolase From the Marine Bacterium Pseudomonas aestusnigri – Structural and Functional Insights. *Frontiers in Microbiology* **Volume 11 - 2020** (2020). https://doi.org:10.3389/fmicb.2020.00114

8 Adams, P. D. *et al.* The Phenix software for automated determination of macromolecular structures. *Methods* **55**, 94-106 (2011). https://doi.org:10.1016/j.ymeth.2011.07.005

9 Emsley, P., Lohkamp, B., Scott, W. G. & Cowtan, K. Features and development of Coot. *Acta Crystallogr D Biol Crystallogr* **66**, 486-501 (2010). https://doi.org:10.1107/s0907444910007493

10 DeLano, W. L. Pymol: An open-source molecular graphics tool. *CCP4 Newsl. protein crystallogr* **40**, 82-92 (2002).

11 Abraham, M. *et al.* (2024).

12 Huang, J. *et al.* CHARMM36m: an improved force field for folded and intrinsically disordered proteins. *Nature Methods* **14**, 71-73 (2017). https://doi.org:10.1038/nmeth.4067

13 Hess, B., Bekker, H., Berendsen, H. J. C. & Fraaije, J. G. E. M. LINCS: A linear constraint solver for molecular simulations. *Journal of Computational Chemistry*

18, 1463-1472 (1997). https://doi.org:https://doi.org/10.1002/(SICI)1096-987X(199709)18:12<1463::AID-JCC4>3.0.CO;2-H

14 Eiamthong, B. *et al.* Discovery and Genetic Code Expansion of a Polyethylene Terephthalate (PET) Hydrolase from the Human Saliva Metagenome for the Degradation and Bio-Functionalization of PET. *Angewandte Chemie International Edition* **61**, e202203061 (2022). https://doi.org:https://doi.org/10.1002/anie.202203061

15 Abid, U. *et al.* Evaluation of enzymatic depolymerization of PET, PTT, and PBT polyesters. *Biochemical Engineering Journal* **199**, 109074 (2023). https://doi.org:https://doi.org/10.1016/j.bej.2023.109074
